# Interferon-γ induces combined pyroptotic angiopathy and APOL1 expression in human kidney disease

**DOI:** 10.1016/j.celrep.2024.114310

**Published:** 2024-06-04

**Authors:** Benjamin A. Juliar, Ian B. Stanaway, Fumika Sano, Hongxia Fu, Kelly D. Smith, Shreeram Akilesh, Suzie J. Scales, Jamal El Saghir, Pavan K. Bhatraju, Esther Liu, Johnson Yang, Jennie Lin, Sean Eddy, Matthias Kretzler, Ying Zheng, Jonathan Himmelfarb, Jennifer L. Harder, Benjamin S. Freedman

**Affiliations:** 1 Division of Nephrology, Department of Medicine, University of Washington School of Medicine, Seattle, WA 98109, USA; 2 Kidney Research Institute, University of Washington School of Medicine, Seattle, WA 98109, USA; 3 Institute for Stem Cell and Regenerative Medicine, University of Washington School of Medicine, Seattle, WA 98109, USA; 4 Division of Hematology, Department of Medicine, Seattle, WA 98109, USA; 5 Department of Bioengineering, University of Washington School of Medicine, Seattle, WA 98109, USA; 6 Bloodworks Northwest Research Institute, Seattle, WA 98102, USA; 7 Plurexa, Seattle, WA 98109, USA; 8 Department of Laboratory Medicine and Pathology, University of Washington School of Medicine, Seattle, WA 98109, USA; 9 Department of Immunology, Genentech, 1 DNA Way, South San Francisco, CA 94080, USA; 10 Division of Nephrology, Department of Internal Medicine, and Department of Computational Medicine and Bioinformatics, University of Michigan Medical School, Ann Arbor, MI 48109, USA; 11 Division of Pulmonary, Critical Care and Sleep Medicine, University of Washington School of Medicine, Seattle, WA 98109, USA; 12 Division of Nephrology and Hypertension, Department of Medicine, Feinberg School of Medicine, Northwestern University, Chicago, IL 60611, USA; 13 Lead contact

## Abstract

Elevated interferon (IFN) signaling is associated with kidney diseases including COVID-19, HIV, and apolipoprotein-L1 (APOL1) nephropathy, but whether IFNs directly contribute to nephrotoxicity remains unclear. Using human kidney organoids, primary endothelial cells, and patient samples, we demonstrate that IFN-γ induces pyroptotic angiopathy in combination with APOL1 expression. Single-cell RNA sequencing, immunoblotting, and quantitative fluorescence-based assays reveal that IFN-γ-mediated expression of APOL1 is accompanied by pyroptotic endothelial network degradation in organoids. Pharmacological blockade of IFN-γ signaling inhibits APOL1 expression, prevents upregulation of pyroptosis-associated genes, and rescues vascular networks. Multiomic analyses in patients with COVID-19, proteinuric kidney disease, and collapsing glomerulopathy similarly demonstrate increased IFN signaling and pyroptosis-associated gene expression correlating with accelerated renal disease progression. Our results reveal that IFN-γ signaling simultaneously induces endothelial injury and primes renal cells for pyroptosis, suggesting a combinatorial mechanism for APOL1-mediated collapsing glomerulopathy, which can be targeted therapeutically.

## In brief

Juliar et al. address interferon signaling in kidney disease. Organoids, primary cells, and clinical datasets reveal that interferon signaling simultaneously induces APOL1 expression and endothelial cell pyroptosis. This suggests a combinatorial mechanism for APOL1-mediated collapsing glomerulopathy, which can be targeted therapeutically. The findings may also be relevant in other organs.

## Graphical Abstract

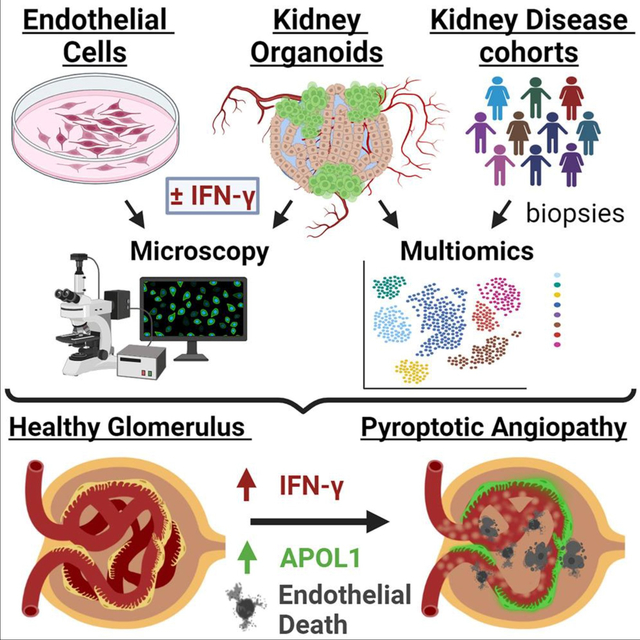

## INTRODUCTION

Maladaptive inflammation is increasingly recognized as an important mediator of disease in diverse solid organ systems such as the kidneys.^1–4^ Elevated levels of interferons (IFNs), circulating cytokines that orchestrate inflammation by inducing expression changes in response genes,^5,6^ are associated with various kidney disease states including lupus nephritis and HIV-associated nephropathy (HIVAN).^7–10^ Therapeutic administration of IFNs can injure kidney glomeruli^11–13^; however, the mechanisms by which IFNs adversely affect kidney epithelial and endothelial cells remain unclear.

One IFN response gene known to be associated with kidney disease is *APOL1*, encoding apolipoprotein-L1 (APOL1), a secreted member of a larger family of apolipoproteins known to modulate immune responses.^14^ Coding variants in *APOL1* common among individuals of recent African ancestry confer a heterozygous advantage against sleeping sickness, but individuals harboring biallelic variants are at increased risk for developing glomerular disease.^15,16^ This appears to be triggered by conditions such as HIV, severe acute respiratory syndrome coronavirus 2 (SARS-CoV-2), hypertension, and lupus,^17,18^ each of which is associated with chronic systemic inflammation and a sustained increase in IFN gene expression.^19–24^ APOL1 is expressed in numerous cell types after IFN exposure,^25,26^ with podocytes believed to be most vulnerable to risk-variant-induced cytotoxicity,^27–33^ although effects have also been observed in endothelial cells (ECs).^34,35^ Various mechanisms of APOL1-mediated cytotoxicity have been proposed^36^; however, recent research has suggested a link to NLRP3-inflammasome activation and pyroptotic cell death.^29,34^

The *APOL1* gene is conserved only among humans and certain non-human primates, with only humans known to have risk variants.^37^ Thus, studies have frequently relied upon exogenous overexpression in animals or cells. For diseases such as APOL1 nephropathy, where a natural animal model does not exist, human organoids provide a valuable surrogate for human-specific responses and regulatory consideration. Kidney organoids derived from pluripotent stem cells contain segments resembling podocytes, proximal tubules, and distal tubules, as well as ECs.^38,39^ Their diverse cell populations and 3D architecture allow for a direct assessment of cell-type-dependent differences in morphological and gene-profiling assays that model disease phenotypes, including settings of viral infection.^40–43^ The treatment of organoids with IFN-γ is associated with increased expression of APOL1 in many cell types, which has been linked to risk-variant-associated cytotoxicity^24^ and the dedifferentiation of podocytes.^40^

Beyond APOL1 induction, the effects of IFN-γ are complex and present a confounding variable in studying kidney disease. Whether IFNs directly contribute to nephrotoxicity, and which specific cell types are involved in this process, remains unclear. Here, we set out to study the larger roles of IFN-γ in kidney organoids and ECs, aligned with urinary and biopsy samples from human patients, to better understand the effects of IFNs on kidneys and potentially other organs.

## RESULTS

### APOL1 is expressed in human kidney tissue and IFN-γ-treated organoids

**Figure 1.**
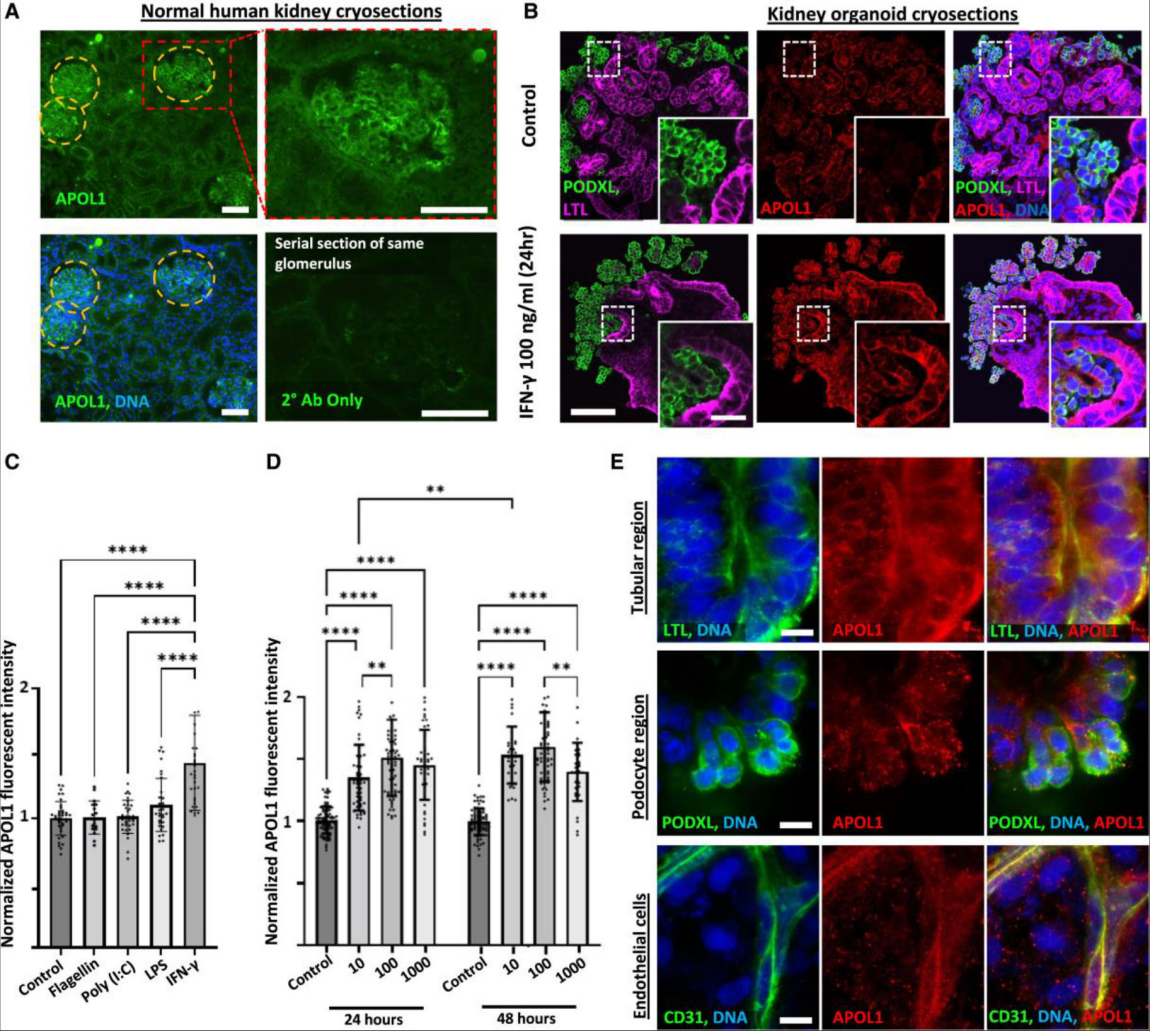
APOL1 is detected by immunofluorescence in IFN-γ-stimulated kidney organoids (A) Disk spinning confocal immunofluorescent images of APOL1 expression in representative serial cryosections of a normal healthy human kidney wedge stained with or without APOL1 primary antibody. Scale bars, 100 μm. (B) Disk spinning confocal immunofluorescence images of representative control and IFN-γ-stimulated organoid cryosections showing APOL1 expression pattern. Scale bar, 100 μm; inset scale bar, 40 μm (C and D) Average fluorescence intensity of APOL1 throughout organoids, quantified with (C) immunofluorescence microscopy for an agonist screen at 24 h (*n* ≥ 17 organoids per condition pooled across 3 independent experiments) and (D) IFN-γ dose response at 10, 100, and 1,000 ng/mL at 24 and 48 h (*n* ≥ 32 organoids per condition pooled across 3 independent experiments). Results are presented as normalized fluorescent intensity. Mean ± SD. Significance was calculated using one-way ANOVA with Tukey’s multiple comparisons test. *p < 0.05, **p < 0.01, and ****p < 0.0001. (E) Laser confocal immunofluorescence images showing subcellular localization of APOL1 expression in tubules, podocytes, and ECs after IFN-γ stimulation. Scale bars, 10 μm. LPS, lipopolysaccharide; CD31, cluster of differentiation 31.

To characterize the timing and cell-type-specific localization of IFN-γ-mediated APOL1 upregulation in our organoid model, we conducted immunofluorescence analysis using a specific antibody recognizing APOL1 (4.17A5).^44,45^ In healthy human kidney cryosections, a strong APOL1 signal was detected in glomerular regions and a weaker signal in tubular regions (Figure 1A). Similarly, in cryosections of human kidney organoids (wild type) in the control condition, a weak signal for APOL1 was detected in both podocyte clusters and proximal tubular structures, identified by co-staining with podocalyxin (PODXL) and *Lotus tetragonolobus* lectin, respectively, with a similar increase in APOL1 immunofluorescence in both nephron segments after IFN-γ treatment (Figure 1B).

In addition to IFNs, Toll-like receptor (TLR) agonists have been shown to upregulate APOL1 expression in various cell types.^46^ Whereas IFN-γ (100 ng/mL) significantly upregulated APOL1 in organoids after 24-h treatment (~50% increase above baseline), the TLR agonists flagellin (1 μg/mL, TLR5), polyinosinic-polycytidylic acid (20 μg/mL, TLR3), and lipopolysaccharide (1 μg/mL, TLR4) had no significant effect (Figure 1C). We compared concentrations ranging from 10 to1,000 ng/mL of IFN-γ and treatment times of 24 h vs. 48 h to determine a dosing regimen that maximized APOL1 expression. 10 ng/mL IFN-γ elicited submaximal APOL1 expression after 24 h, whereas 100 ng/mL IFN-γ provided a saturating dose with plateauing signal from 24 to 48 h (Figure 1D). Following 24-h IFN-γ (100 ng/mL) stimulation, the APOL1 signal was primarily localized to membrane compartments, with faint diffuse cytoplasmic staining, and brighter, more focal outer-cell-membrane staining of podocytes, tubular cells, and ECs (Figure 1E).

### JAK1/2 inhibitors block and revert IFN-γ-induced APOL1 expression

**Figure 2.**
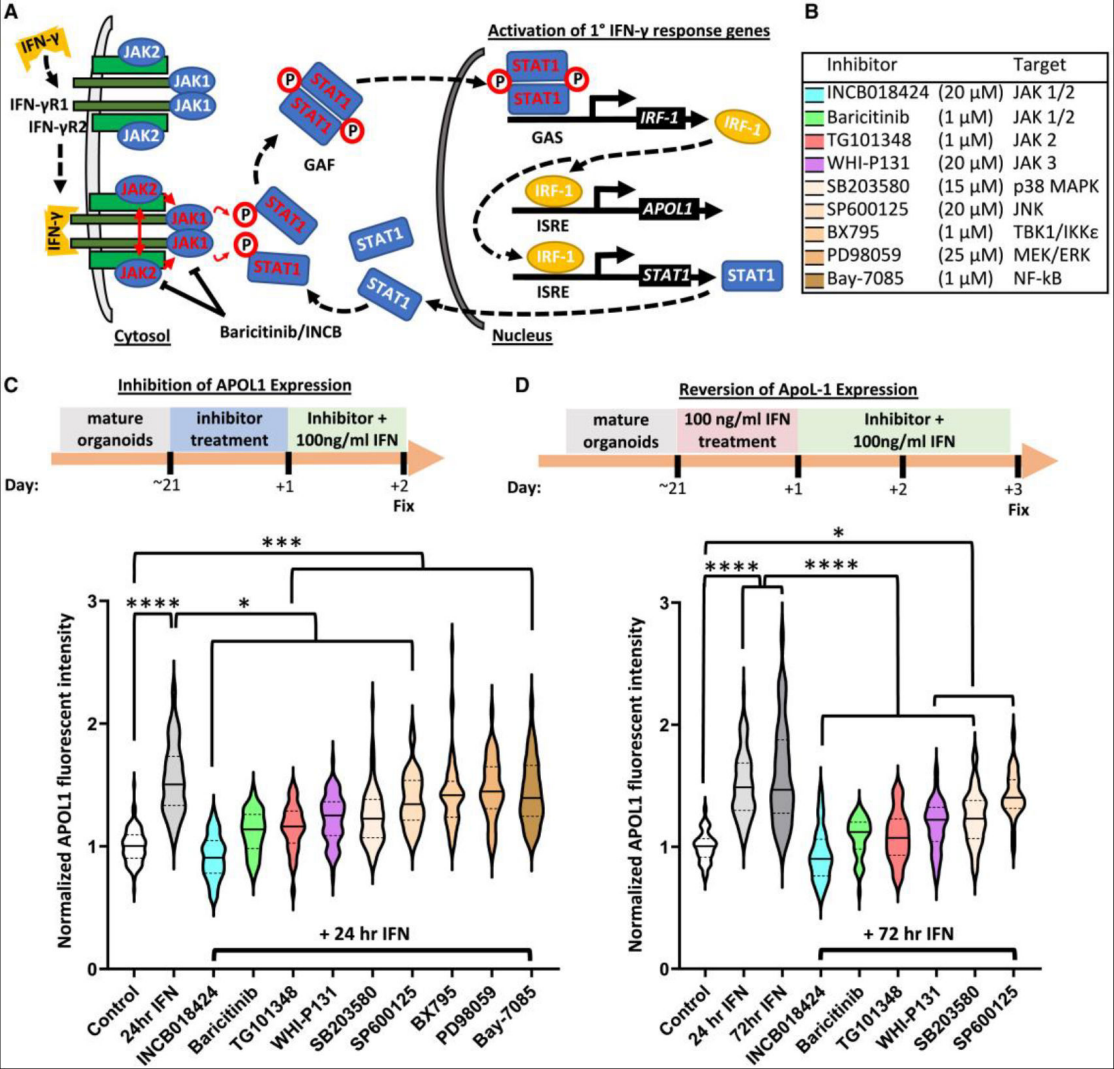
JAK1/2 inhibitors prevent and reverse IFN-γ-mediated APOL1 expression (A) Cartoon illustrating canonical IFN-γ signaling and APOL1 induction and JAK1/2 inhibition by baricitinib and INCB018424. Red text indicates phosphorylation and activation. IFN-γR, interferon-γ receptor; JAK, Janus kinase; STAT, signal transducer and activator of transcription; GAF, gamma-interferon activation factor; GAS, gamma-interferon activation site; IRF, interferon regulatory factor; ISRE, interferon stimulated response element. (B) Legend for drug screen, including drug target. (C and D) Dosing regimens starting on day 21 of organoid culture are indicated above violin plots of APOL1 signal intensity to test prevention (*n* ≥ 44 organoids per condition pooled across 6 independent experiments) (C) and reversion (*n* ≥ 26 organoids per condition pooled across 5 independent experiments) of IFN-γ induced APOL1 expression (D). Lines indicate median (solid) and quartiles (dashed). Significance was calculated using one-way ANOVA with Tukey’s multiple comparisons test. *p < 0.05, ***p < 0.001, and ****p < 0.0001.

Canonical IFN-γ-mediated signaling proceeds through Janus kinase (JAK) activation of the signal transducer and activator of transcription (STAT) signaling pathway, primarily initiated by the JAK1 and JAK2 subunits of the IFN-γ receptor (Figure 2A).^47,48^ Nine pharmacological compounds targeting JAK1–3 and various intracellular mediators of the innate immune response were tested in repeated experiments (Figure 2B). When added prior to IFN-γ, several of these compounds significantly reduced the APOL1 signal relative to IFN-γ treatment (Figure 2C). The clinically approved JAK1/2 inhibitors INCB018424 (ruxolitinib) and baricitinib had the greatest effects, which were statistically indistinguishable from the unstimulated control (Figure 2C).

In a second series of experiments, effective compounds from the first screen were tested following 24-h IFN-γ treatment to determine whether these compounds could rescue IFN-γ-induced APOL1 expression. All but one of these compounds also exhibited therapeutic efficacy in this scenario (Figure 2D). JAK1/2 inhibition with INCB018424 and baricitinib, as well as specific JAK2 inhibition with TG101348, returned the APOL1 expression to baseline (Figure 2D). These results demonstrated the capacity to inhibit IFN-γ induction of APOL1 pharmacologically and supported a mechanistic role of canonical JAK-STAT signaling underlying this induction.

### IFN-γ induces pyroptosis-associated genes across organoid cell types

**Figure 3.**
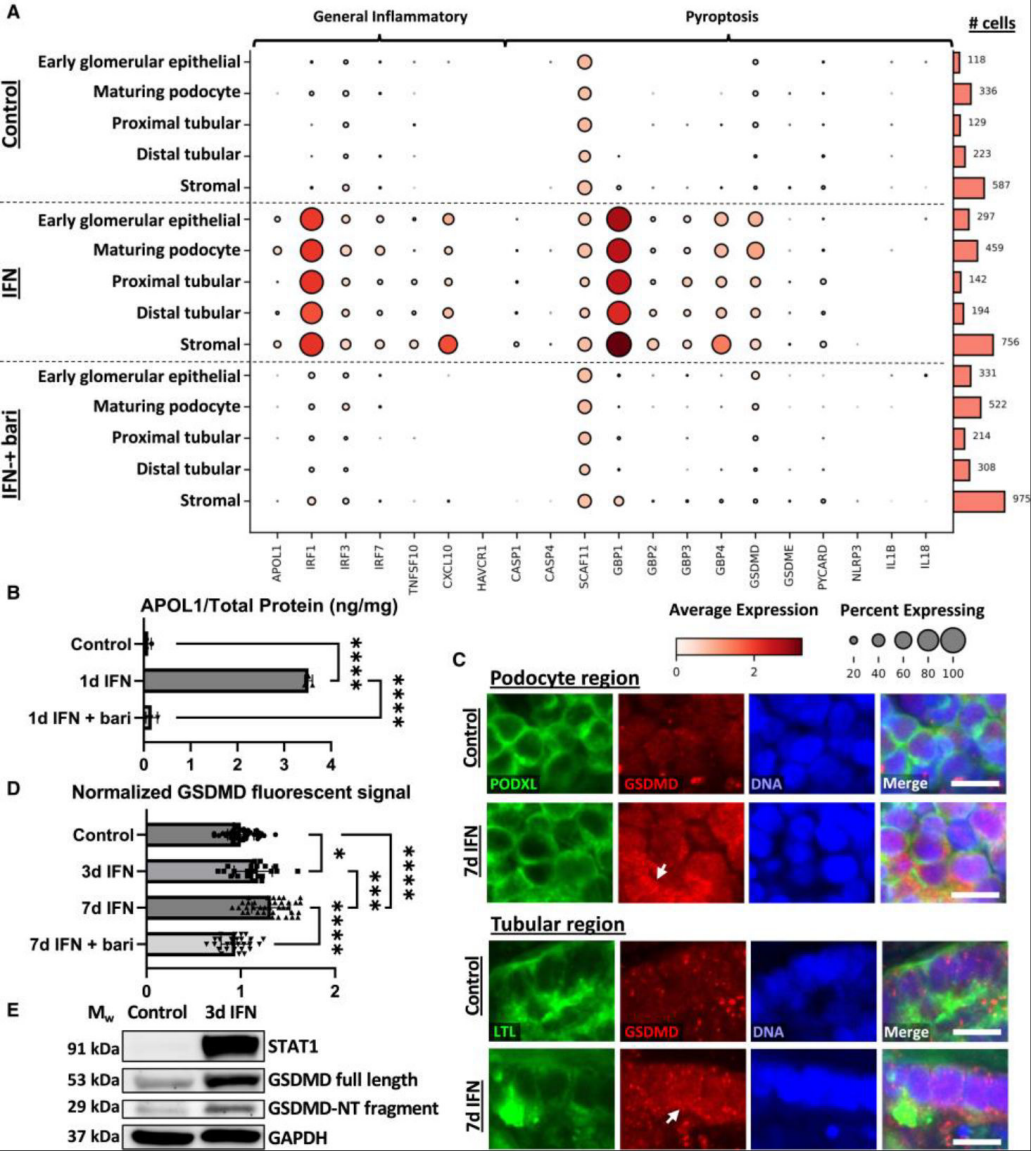
IFN-γ stimulation induces key pyroptosis-associated genes across cell types (A) Dot plot of inflammatory and pyroptosis-associated gene expression in organoids treated with 25 ng/mL IFN-γ for 24 h ± 1 μM baricitinib (bari). Darker dots indicate stronger expression, and dot size reflects the percentage of cells in a cluster expressing the indicated gene. Cell counts per type, per condition, are indicated to the right. (B) ELISA showing APOL1 expression in organoids treated with 25 ng/mL IFN-γ for 24 h ± 1 μM baricitinib (*n* = 3 independent experiments). (C) Confocal immunofluorescence images showing GSDMD signal in both podocytes (top) and tubules (bottom) with punctate densities localized to the outer cell membrane (white arrows). Scale bars, 10 μm. (D) Average GSDMD signal throughout the organoid following daily treatment with 100 ng/mL IFN-γ ± 1 μM baricitinib, quantified with immunofluorescence microscopy (*n* ≥ 22 organoids per condition pooled across 4 independent experiments). Mean ± SD. Significance was calculated using one-way ANOVA with Tukey’s multiple comparisons test. *p < 0.05, **p < 0.01, ***p < 0.001, and ****p < 0.0001. (E) Representative western blot from bulk lysate of organoid cultures showing STAT1, full-length GSDMD, and the N-terminal (NT) fragment of GSDMD with 3 day IFN treatment. See also Figure S1.

To gain insight into injury and therapeutic states associated with these treatments, we analyzed single-cell RNA sequencing (scRNA-seq) of organoids treated ± IFN-γ ± baricitinib (1 μM) for 24 h for signatures of programmed cell death. 25 ng/mL IFN-γ was used for these experiments, which resulted in a similar increase in APOL1 immunofluorescent signal to 100 ng/mL (Figure S1A). No changes in apoptosis-, necroptosis-, or ambiguous programmed cell death-associated gene expression were detected in any organoid cell type (Figure S1B). In contrast, marked changes in inflammatory and pyroptosis-associated genes^49^ were detected, including IFN response factors (*IRF1,3,7*), *TNFSF10*, *CXCL10*, caspase-1 (*CASP1*), gasdermin-D (*GSDMD*), and guanylate-binding proteins (*GBP1,2,3,4*), across diverse organoid cell types including podocytes, tubular epithelial cells, and stromal cells (Figure 3A). *GSDMD*, a primary end mediator of pyroptotic cell swelling and lysis when cleaved,^49^ exhibited the highest expression levels in maturing podocytes (Figure 3A). Inclusion of baricitinib during IFN-γ treatment reduced pyroptosis-associated gene expression to baseline (Figure 3A). ELISAs performed on protein lysates from organoid cultures similarly demonstrated an increase in APOL1 expression, which could be inhibited with baricitinib (Figure 3B).

Immunofluorescent microscopic analysis of GSDMD protein was conducted to investigate protein expression in the context of altered gene expression. Consistent with scRNA-seq, organoids treated daily with IFN-γ (100 ng/mL) for 7 days showed an increase in GSDMD signal in both podocyte and tubular regions, with GSDMD generally appearing throughout cell bodies with occasional punctate densities along their outer cell membranes (Figure 3C). The progressive increase in average GSDMD signal throughout the organoid could be inhibited by the inclusion of baricitinib (1 μM) (Figure 3D). Quantitative western blot analysis further confirmed the significant upregulation of full-length GSDMD, cleavage of GSDMD to produce the N-terminal GSDMD fragment, and upregulation of STAT1, a marker of IFN signaling (Figures 3E and S1C–S1I). Collectively, these findings revealed upregulation of pyroptotic signatures with IFN-γ treatment, suggesting that IFN-γ may result in kidney cells undergoing pyroptotic cell death.

### Podocytes are resilient to prolonged IFN-γ treatment

**Figure 4.**
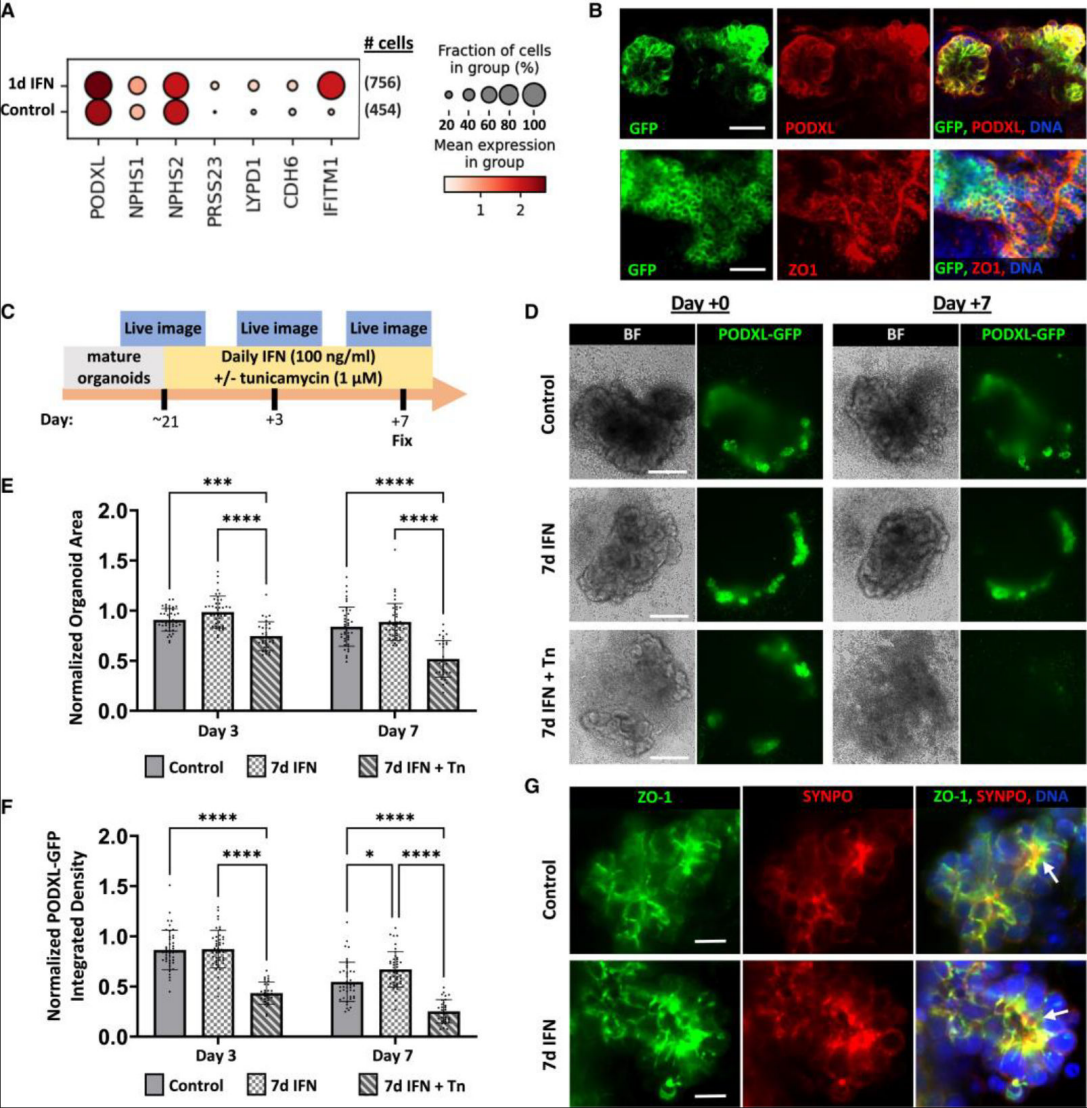
Podocytes are resilient to prolonged IFN-γ treatment despite modest dedifferentiation (A) Dot plot of mature and early glomerular epithelial gene expression from scRNA-seq in pooled mature and early glomerular cell fractions from organoids treated with 25 ng/mL IFN-γ for 24 h. Darker red dots indicate stronger expression across cells, and dot size reflects the percentage of cells expressing the indicated gene. (B) Confocal immunofluorescence images of PODXL-GFP organoids stained for PODXL or ZO-1. Scale bars, 40 μm. (C) Timeline for prolonged IFN-γ treatment and live imaging of PODXL-GFP organoids. (D) Representative bright-field images and maximum intensity projections of disk spinning confocal z stacks from live imaging. Scale bars, 200 μm. (E and F) Quantification of organoid areas (E) and quantification of PODXL-GFP fluorescent signal integrated density normalized to pretreatment (F) (*n* ≥ 24 organoids per condition pooled across 4 independent experiments). Mean ± SD. Significance was calculated using one-way ANOVA with Tukey’s multiple comparisons test. *p < 0.05, ***p < 0.001, and ****p < 0.0001. (G) Representative laser confocal z-slices showing co-localization of ZO-1 and SYNPO in podocyte clusters after prolonged IFN-γ treatment. Scale bars, 10 μm. ZO-1, zonula occludens-1; SYNPO, synaptopodin; Tn, tunicamycin. See also Figure S2.

As podocytes are hypothesized to be highly vulnerable in APOL1-associated nephropathy, we investigated kidney organoid podocytes for evidence of injury associated with IFN-γ treatment. By single-cell transcriptional profiling, we assessed the expression of early and mature podocyte markers in the pooled population of early glomerular epithelial cells (EGEs) and mature podocytes. Increased expression of *PRSS23*, *CDH6*, and *LYPD1* with IFN-γ treatment, which also robustly induced the IFN-induced transmembrane protein 1, suggested a shift in the total podocyte population toward EGE gene expression and possible dedifferentiation relevant to glomerular disease (Figure 4A).^50,51^

To assess whether this transcriptional signature of dedifferentiation corresponded with morphological changes, we developed a live imaging assay tracking podocyte clusters within individual organoids. To visualize podocytes, we generated induced pluripotent stem cell (iPSC) lines expressing endogenous PODXL tagged on the C terminus with green fluorescent protein (PODXL-GFP) (Figures S2A–S2C). In these iPSC clones, in the undifferentiated state, a GFP band was specifically observed at ~250 kDa by immunoblot, which overlapped with PODXL bands (Figures S2D and S2E). Using live confocal microscopy, PODXL-GFP was readily detected in a bright punctate pattern decorating the apical surface of iPSCs and was enriched at cell-cell junctions and the ciliary necklace, precisely matching PODXL antibody immunofluorescence (Figures S2F and S2G). During organoid differentiation, PODXL-GFP was initially expressed at the luminal surface of epiblast spheroids, subsequently downregulated during the induction of nephron progenitor cell differentiation, and ultimately re-expressed in bright clusters in maturing kidney structures (Figure S2H). Immunofluorescence analysis confirmed that PODXL-GFP strongly co-localized with the PODXL antigen signal at the apical cell surface of organoid podocytes, reciprocal to zonula occludens-1 (ZO-1) at the basal surface (Figures 4B, S2I, and S2J). These findings matched the expected expression pattern of PODXL in iPSCs and organoids.^38,52^

Live time course imaging of kidney organoids expressing PODXL-GFP was used to investigate whether organoids progressively lost podocytes with prolonged IFN-γ treatment. Individual organoids with well-defined podocyte regions were imaged live immediately prior to treatment and after 3 and 7 days (Figure 4C). Tunicamycin treatment was included as a control to induce organoid degradation.^40^ An assessment of bright-field images indicated that control- and IFN-γ-treated organoids both retained well-defined tubular structures over 1 week, whereas the inclusion of tunicamycin induced overt loss of organoid structure and regression (Figure 4D). The quantification of the organoid area indicated no relative differences between control- and IFN-γ-treated groups over time, but there was a progressive decrease in total organoid area and loss of podocyte tufts with the inclusion of tunicamycin (Figure 4E). Whereas the size of podocyte tufts in any given organoid remained similar over time in control- and IFN-γ-treated conditions, a decrease in PODXL-GFP signal intensity was observed in all conditions and was rapid and severe in tunicamycin-treated organoids (Figures 4D–4F). IFN-γ-treated organoids maintained a slightly higher PODXL-GFP signal relative to control on day 7 (Figure 4F). Immunofluorescence analysis of organoid podocytes exposed to IFN-γ for 7 days revealed normal co-localization of the slit diaphragm components ZO-1 and synaptopodin at basal junctional complexes (Figure 4G).^38,52^ Collectively, these results surprisingly suggested no loss or disruption of podocyte clusters or tubular structures over time with 7 days of IFN-γ treatment.

### Prolonged IFN-γ treatment induces endothelial network degradation

**Figure 5.**
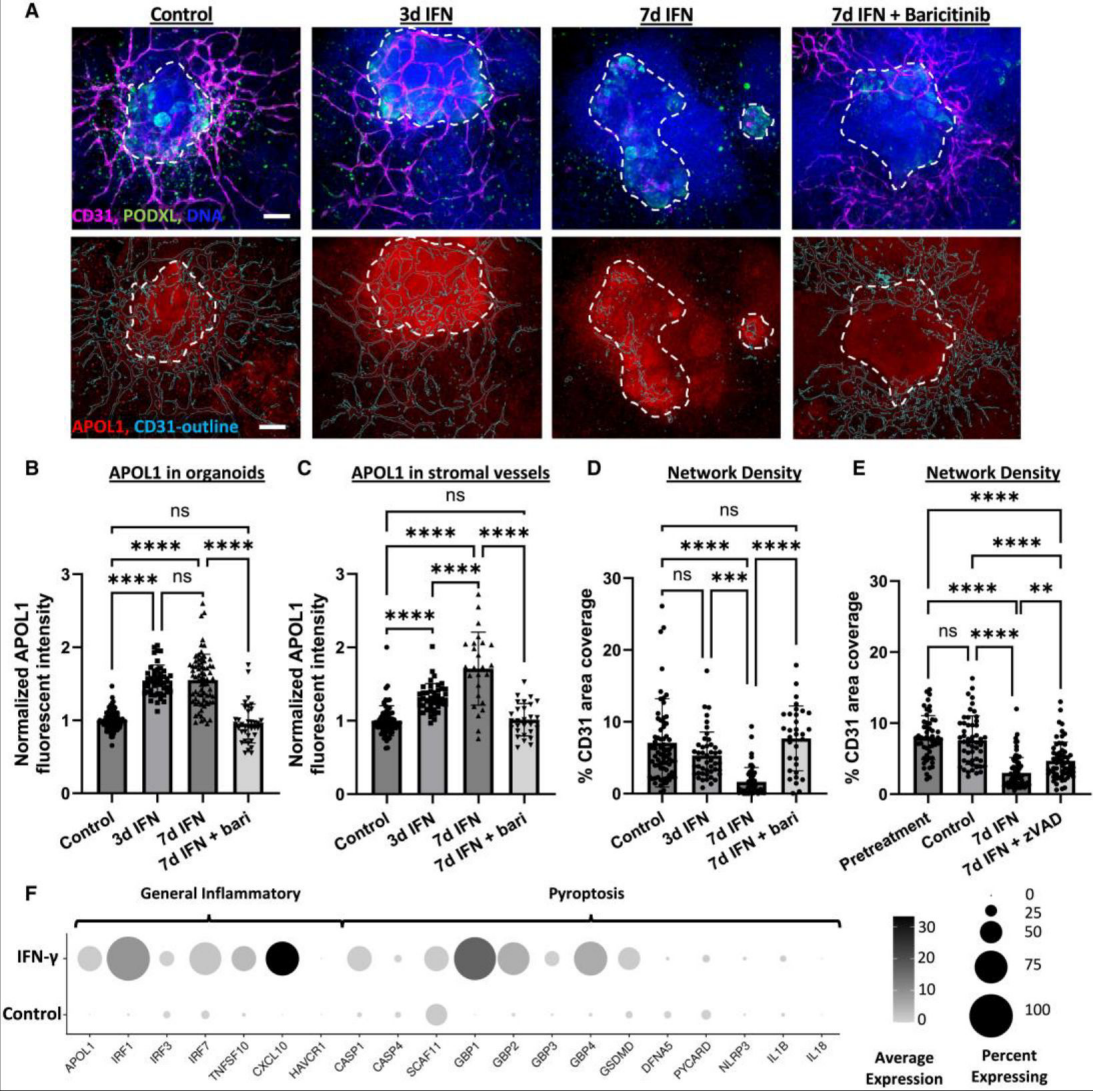
IFN-γ-induced degradation of endothelial networks is rescued by JAK1/2 inhibition and partially rescued by caspase inhibition (A) Representative maximum intensity projections of disk spinning confocal z stacks. Manual trace of the organoid body (white dashes) and automated trace of vascular network (teal, lower row) are superimposed. Scale bars, 100 μm. (B) Average fluorescent intensity of APOL1 throughout organoids quantified with immunofluorescence microscopy (*n* ≥ 37 organoids between 4 independent experiments). (C) Average fluorescent intensity of APOL1 within CD31^+^ binary mask, excluding organoid body manual trace (stromal area), quantified with immunofluorescence microscopy (organoids with stromal network areas ≤2,000 μm^2^ were excluded, *n* ≥ 24 organoids/condition pooled between 4 independent experiments). (D and E) Automated quantification of total CD31^+^ network density per region of interest containing an organoid for (D) baricitinib (*n* ≥ 32 organoids per condition pooled between 4 independent experiments) and (E) zVAD-FMK (*n* ≥ 52 organoids per condition pooled between 4 independent experiments) rescue experiments. Mean ± SD. Significance was calculated using one-way ANOVA with Tukey’s multiple comparisons test. *p < 0.05, **p < 0.01, ***p < 0.001, and ****p < 0.0001. (F) Dot plot of gene expression in ECs from organoids treated with 25 ng/mL IFN-γ for 24 h. Darker dots indicate stronger expression across cells, and dot size reflects the percentage of cells expressing the indicated gene. See also Figures S3 and S4 and Table S1.

We further investigated the effect of IFN-γ treatment on ECs, which are essential components of renal glomeruli and naturally form networks alongside epithelial structures in kidney organoids.^38,39^ Prolonged IFN-γ exposure resulted in a pronounced loss of the endothelial network surrounding organoids, as indicated by the loss of CD31^+^ networks (Figure 5A) and co-localized PODXL signal (Figure S3A). The APOL1 signal intensity in endothelial vessels surrounding organoids gradually increased during 7 days of IFN-γ treatment, in contrast to neighboring epithelial structures in which the APOL1 signal plateaued after the third day (Figures 5B and 5C). The quantification of total endothelial network density demonstrated no significant loss of endothelial networks after 3 days but a pronounced loss after 7-day IFN-γ treatment that could be prevented by the inclusion of baricitinib (1 μM) during treatment (Figure 5D). However, the vessel network density within the stromal area surrounding the epithelial organoid structures did significantly drop after 3 days of IFN-γ treatment, suggesting that vessel structures outside the organoids may be more susceptible or responsive to IFN signaling (Figure S3B). Prior to treatment (pretreatment), organoid cultures contained fully formed endothelial networks at a similar density to controls after 7 days, indicating that ECs were lost over time in the treated cultures (Figure 5E). Pan-caspase inhibition with zVAD-FMK (30 μM) significantly prevented IFN-γ-induced endothelial network loss, demonstrating that programmed cell death contributes to endothelial network loss (Figure 5E).

Since ECs were not identified in our current scRNA-seq dataset, we turned to our previously published Liu et al. scRNA-seq dataset of IFN-γ-treated organoids in which a small cluster of ECs were detected to explore gene expression profiles in ECs following IFN-γ treatment (25 ng/mL, 24 h).^40^ Consistent with our newly generated scRNA-seq dataset, this dataset also demonstrated pyroptosis-associated gene upregulation across organoid cell types with IFN-γ treatment and increased upregulation of CASP1 and TNFSF10 in the endothelial fraction (Figures 5F and S3C). To assess the possible impact of altered cellular crosstalk between ECs and other cell types, we also profiled changes in pro- and anti-angiogenic cytokine and receptor expression across cell types. We did not observe any notable changes except for CXCL10, which was prominently upregulated in ECs (Figure S3D).

Gene Ontology (GO) pathway analysis was performed on the Liu et al. scRNA-seq dataset to broadly investigate changes that occur with IFN-γ treatment. The top 20 GO terms for all cell types combined included several terms related to the immune response, enhanced expression of genes in the nuclear factor κB signaling pathway (which plays an upstream role in activating pyroptosis^49^), and changes to the electron transport chain (Figure S4A). When separated into distinct cell clusters, various cell types demonstrated broadly similar changes, but notably, the pyroptosis pathway was more significantly upregulated in the EC cluster (*p* = 2.29E–06), compared to all cell types combined (*p* = 2.03E–05) (Table S1).

As our GO pathway analysis of organoids highlighted changes in aerobic respiration in response to IFN-γ treatment, we further validated this result functionally using a Seahorse assay. To accommodate this assay, we utilized homogeneous cultures of endothelial-like cells derived from iPSCs rather than organoids. These cultures demonstrated an increased oxygen consumption rate and spare respiratory capacity when treated with varying doses of IFN-γ (Figure S4B). Taken together, our findings support the broad upregulation of inflammatory and pyroptosis-associated gene expression across cell types but a specific injurious impact of IFN-γ in ECs that is partially mediated by cell death and accompanied by metabolic changes.

### Primary human AKECs exhibit pronounced sensitivity to IFN-γ

We sought to obtain independent confirmation that the effects we observed in kidney organoid ECs were specific and physiologically relevant to primary human adult kidney ECs (AKECs). To accomplish this, we compared primary AKECs to commercially available human umbilical vein ECs (HUVECs). Selective expression of the fenestration-associated protein, plasmalemmal vesicle associated protein-1 (PV-1) was used to confirm the renal origin of isolated AKECs (Figure S5A).

**Figure 6.**
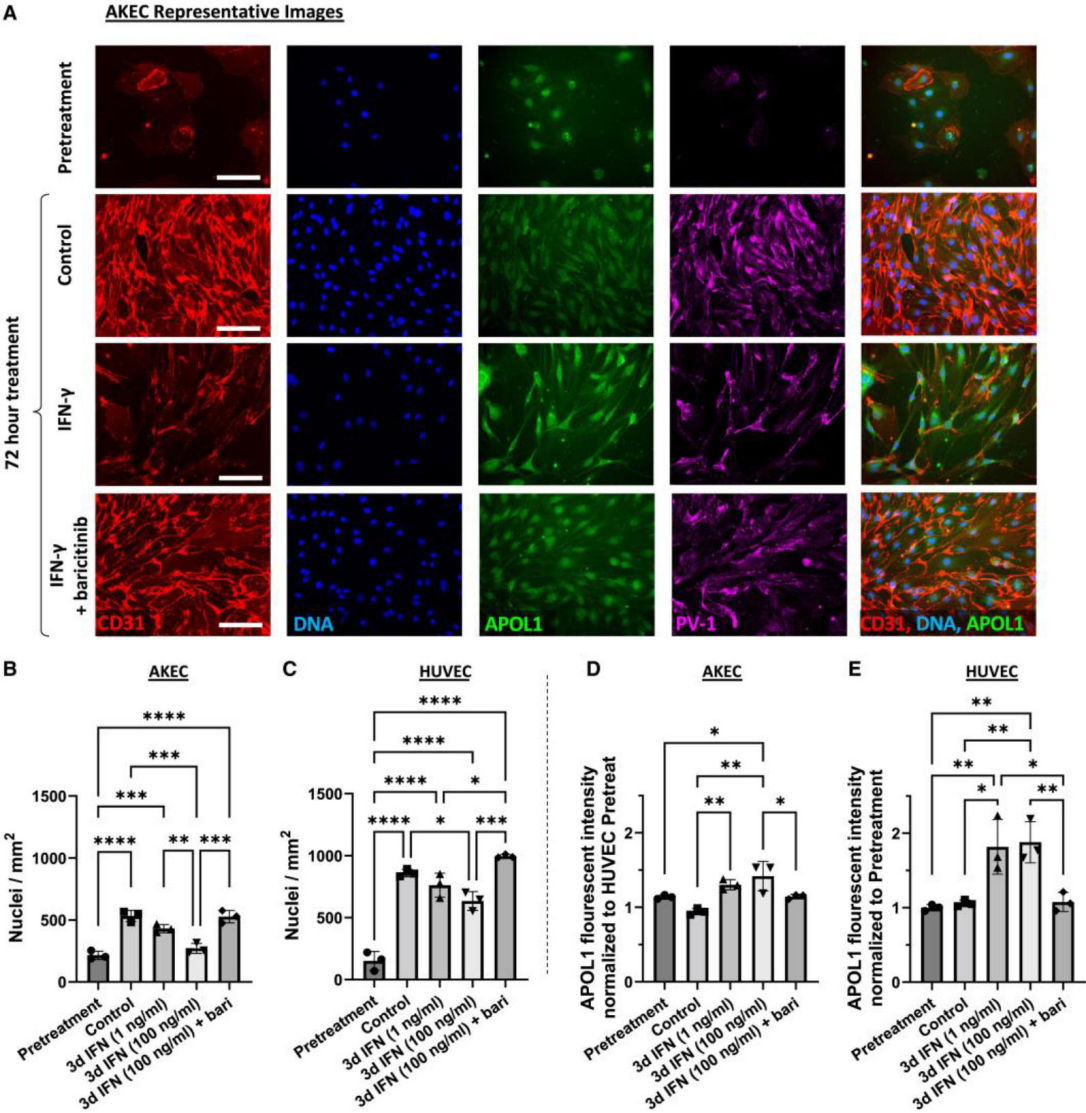
AKECs exhibit pronounced sensitivity to IFN-γ treatment (A) Representative wide-field immunofluorescent images of AKECs 24 h after plating (pretreatment) and 72 h after treatment with IFN-γ (100 ng/mL) ± baricitinib (1 μM). Scale bars, 100 μm. PV-1, plasmalemmal vesicle associated protein-1. (B–E) Automated quantification of (B and C) average nuclear density and (D and E) APOL1 fluorescent intensity in cell bodies during monolayer culture of HUVECs (*n* = 3 independent lots) and primary human AKECs (*n* = 3 independent donors). Mean ± SD. Significance was calculated using one-way ANOVA with Tukey’s multiple comparisons test. *p < 0.05, **p < 0.01, ***p < 0.001, and ****p < 0.0001. See also Figure S5.

When subconfluent cultures were treated with IFN-γ, AKECs exhibited a compact, spindle-like morphology and failed to form confluent monolayers, whereas HUVECs continued to do so (Figures 6A and S5B). Quantitative fluorescence microscopy analysis demonstrated that AKECs treated with 100 ng/mL IFN-γ showed a dramatic dose-dependent decrease in cell number, whereas HUVECs displayed only a modest decrease (Figures 6B and 6C). To gain further insight into the effect of IFN-γ on AKEC proliferation versus cell loss, AKEC cultures were grown to confluence prior to treatment. IFN-γ treatment for 3 days was insufficient to induce loss of nuclei density, but a significant decrease in CD31 immunostaining was detected, which could be partially rescued with baricitinib treatment (Figures S5C–S5E). With regard to APOL1 expression, HUVECs exhibited a robust response, whereas AKECs exhibited a cell-density dependent effect; subconfluent cultures showed only a modest increase after treatment, but confluent cultures demonstrated robust upregulation of APOL1 (Figures 6D, 6E, S5C, and S5F). These results suggested endothelial-origin- and cell-density-dependent differences in the anti-angiogenic effect of IFN-γ, and that ECs of renal origin are particularly sensitive to IFN-γ treatment, likely via an APOL1-in-dependent mechanism.

### IFN signaling and upregulation of pyroptosis-associated genes in patients correlate with accelerated renal failure

**Figure 7.**
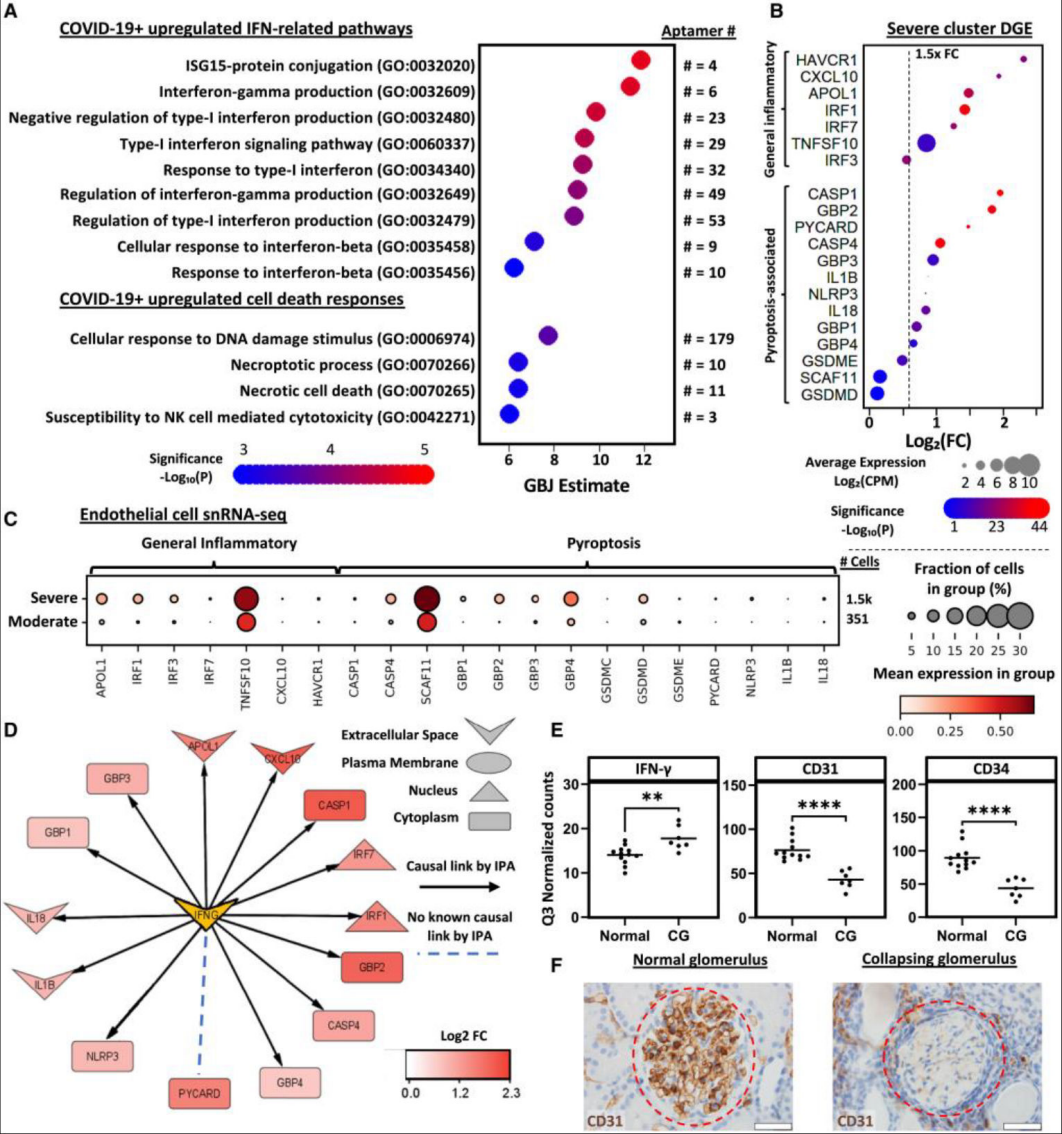
IFN signaling and upregulation of pyroptosis-associated genes in patients correlates with accelerated renal failure (A) Gene Ontology pathway analysis of urine proteome reads showing the Generalized Berk-Jones (GBJ) statistic and significance (adjusted *p* value) for up-regulated IFN- and cell-death-related pathways in COVID+ versus COVID− patients. (B) Differential gene expression (DGE) analysis for inflammatory and pyroptosis associated genes on voom-transformed normalized read counts from bulk RNA-seq on biopsies from individuals in the severe cluster relative to the moderate cluster, showing fold change (FC), significance (adjusted *p* value), and counts per million reads. (C) snRNA-seq showing upregulation of pyroptosis-associated genes in the EC fraction from severe cluster biopsies compared to moderate. (D) IPA wheel spoke diagram showing causal links (black arrows) of IFN-γ as an upstream regulator of pyroptotic genes. Gene shapes indicate cellular localization, and color indicates log2FC from DGE analysis. (E) Third quartile (Q3) normalized counts for *IFN-γ*, *CD31*, and *CD34* from collapsing glomeruli (*n* = 7, from 3 patients) and normal glomeruli (*n* = 12, 3 patients) are shown. Each point represents the expression level of that gene from GeoMx digital spatial profiling for an individual glomerulus. Mean shown. Significance was calculated using a two-tailed t test. **p < 0.01 and ****p < 0.0001. (F) Immunohistochemistry for CD31 in a representative glomerulus with collapsing histology (HIVAN) and another with normal histology (glomerular borders outlined in red). Scale bars, 20 μm. See also Figure S6 and Table S2.

We sought to determine whether similar pathways were activated in diseased human kidneys. We and others have previously identified IFN signaling as a major consequence of SARS-CoV-2 infection.^24,42^ We therefore re-analyzed a urinary proteomics dataset collected from prospectively enrolled critically ill patients with symptoms suggestive of SARS-CoV-2 infection.^42^ GO analysis identified 186 non-redundant terms with a false discovery rate <0.05 (Table S2) in patients with COVID-19 compared to symptom-matched control patients that were found to be negative for COVID-19. COVID-19-positive patients had a 2-fold increased need for renal replacement therapy by the time of hospital discharge,^42^ correlating with the upregulation of type I and II IFN-related and cell death response pathways (Figure 7A). Thus, the dataset supported an association between IFN upregulation, cell death, and kidney disease.

To explore a cohort of patients with glomerular disease, we examined a previously published dataset available from the North American Nephrotic Syndrome Study Network (NEPTUNE) for signatures of pyroptosis-associated gene upregulation depending on the severity of disease progression.^53^ NEPTUNE study participants encompassed patients with biopsy-proven proteinuric kidney disease including minimal change disease or focal segmental glomerulosclerosis (FSGS). Unbiased consensus clustering of gene expression profiles from bulk RNA sequencing of biopsies clustered participants into 3 clusters with shared molecular signatures, one of which was defined by a molecular signature consistent with the high activation of tumor necrosis factor (TNF)-related pathways, corresponding to a significantly increased risk (hazard ratio: 5.23) of reaching a 40% decline in estimated glomerular filtration rate (eGFR) (“severe”) compared to the other two clusters with low TNF pathway activation (“moderate”).^53^ Differentially expressed genes (DEGs) were identified by performing limma^54^ on voom-transformed data^55^ on the severe cluster relative to the other clusters for all 29,188 genes in the dataset. We found that individuals grouped into the severe cluster demonstrated increased expression of numerous pyroptosis associated genes (Figure 7B) but minimal upregulation of apoptosis-associated genes or downregulation of angiogenesis-associated genes (Figure S6). In single-nucleus RNA-seq (snRNA-seq) datasets from biopsies of 5 individuals in the severe cluster compared to 5 in the moderate cluster,^53^ we observed the upregulation of pyroptosis-associated genes specifically in the EC fraction (Figure 7C). Notably, there was also increased expression of *CASP4*, *CASP11* (also known as *SCAF11*), *GBP*s, *PYCARD*, *NLRP3*, and *GSDMD* in ECs compared to podocytes (Figure S6B). However, the magnitude of *PYCARD* and *NLRP3* expression was relatively low, similar to our organoid dataset, suggesting the possibility of non-canonical inflammasome activation that can occur independently of the NLRP3 inflammasome.^49^

We further analyzed these datasets for evidence of IFN-γ involvement. Upstream regulator analysis was performed in ingenuity pathways analysis (IPA) on DEGs in the severe cluster using a fold change > |1.5| and an adjusted *p* < 0.05. As previously reported,53 TNF activation was the top predicted upstream regulator, followed by a predicted activation of IFN-γ (supported by 599 DEGs). Focusing on DEGs involved in pyroptosis, IPA indicated IFN-γ as being upstream of all pyroptosis-associated genes, except there was no known link with *PYCARD* (Figure 7D).

To visualize the effects of inflammatory kidney disease on ECs *in vivo*, we analyzed a digital spatial profiling dataset of collapsed glomeruli in patients with COVID-19 or HIV compared to histologically normal glomeruli from patients presenting with hematuria.^56^ Collapsing glomeruli showed significantly increased expression of *IFN-γ*, *PYCARD*, *GBP1*, and *IL18* and reduced *CD31* and *CD34* expression (Figures 7E and S6C). Immunohistochemical analysis similarly demonstrated reduced CD31 staining in collapsed glomeruli at the protein level, indicating injury or loss of the endothelium, with no apparent immune cell infiltration (Figure 7F).

Collectively, our results from multiple cohorts demonstrate IFN activity and EC-specific pyroptotic injury profiles in kidney disease in settings of APOL1 upregulation. Together with our analyses *in vitro*, these results may suggest a widespread involvement of pyroptotic angiopathy concurrent with APOL1 upregulation, possibly contributing to a synergistic feedforward effect of glomerular sclerosis and collapse.

## DISCUSSION

### Direct effects of IFNs beyond APOL1

Although IFN-γ is strongly associated with APOL1 expression in podocytes, the broader effects of IFN-γ on kidney cell types has been difficult to discern. Our results suggest that IFN-γ has additional effects on kidney cell types beyond increasing APOL1 expression, which could contribute directly to pathophysiology. In particular, we observe a dramatic injurious effect of IFN-γ on ECs in both iPSC-derived organoids and primary cultures from adult kidneys. In the organoid model, IFN-γ stimulation upregulated pyroptosis-associated genes including *CASP1*, *GBP1–4*, *PYCARD*, and *GSDMD* across cell types. These genes help mediate the cleavage of GSDMD, which we observed in organoids. Cleaved GSDMD forms cell membrane pores mediating pyroptotic cell swelling and lysis.^49^

The broad upregulation of pyroptosis-associated genes in IFN-treated organoids was not associated with overt morphological changes in organoid podocyte or tubular regions but was associated with a dramatic loss of endothelial networks that was caspase dependent. Single-cell transcriptional profiling of organoids treated with IFN-γ demonstrates pronounced *CASP1*, *CXCL10*, and *TNFSF10* upregulation in ECs compared to other organoid cell types. CXCL10 is an inflammatory cytokine with diverse paracrine and autocrine functions including immune cell chemoattraction and inhibition of proliferation and migration,^57^ while TNFSF10 evokes cell stress pathways that can mediate cell death through either apoptotic or pyroptotic mechanisms.^58^ This may reflect a greater sensitivity of ECs to inflammatory signaling, injury, and cell death versus epithelial lineages^59^ or may relate to differences in cell death pathways depending on cell type.^29,60^ The analysis of primary cells also suggests that kidney ECs may be particularly sensitive to IFN-γ signaling with a complete inhibition of proliferation, changes in aerobic respiration, and reduced CD31 expression. These effects on ECs in combination with APOL1-mediated podocytopathy may synergistically contribute to kidney disease.

Although various cell death mechanisms may contribute to kidney injury,^61^ our analysis of kidney tissue data from individuals with proteinuric kidney disease shows that broad upregulation of pyroptosis-associated, but not apoptosis-associated, genes correlates with worsened disease prognosis in kidney disease. These upregulated genes include *CASP1*, *CASP4*, *PYCARD*, *NLRP3*, and *GBPs* in bulk RNA-seq from kidney biopsies. Each of these genes, as well as *CASP11* and *GSDMD*, were also upregulated specifically in the EC cluster from snRNA-seq. Moreover, we observed a dramatic loss of endothelium in collapsed glomeruli concurrent with increased *IFN-γ*, *PYCARD*, *GBP1*, and *IL18* expression. Infiltrating immune cells were not observed in these glomeruli. These findings are consistent with the hypothesis that circulating plasma factors cause glomerular cell damage preceding primary FSGS, and a direct interaction with immune cells is unknown.^62^ We also identified IFN-γ as an upstream regulator of these DEGs in diseased kidney tissue. The upregulation of IFN-signaling pathways in COVID-19 patients with an increased need for renal replacement therapy further supports the contribution of IFN signaling in kidney disease pathology and the relevance of our organoid-based model.

Notably, in patients with chronic kidney disease, microvascular rarefaction is observed in other organ systems, which may reflect a role for systemic inflammation involving IFNs and other pathways.^63^ Despite potential physiological distinctions, mouse models have helped elucidate the involvement of inflammatory cell death in kidney disease. Mice with cisplatin-induced kidney injury show increased GSDMD protein expression localized to the peritubular region,^64^ agreeing with our observation of pronounced *GSDMD* expression in renal ECs. Transgenic expression of risk variant *APOL1* in mouse podocytes has also been shown to induce a pyroptotic signature and fibrosis, even in the absence of IFN administration, which was dependent on GSDMD and NLRP3.^29^ However, contradictory experiments in mice and human podocyte cell lines have also drawn into question the existence of the NLRP3 inflammasome in kidney epithelial cells.^64,65^ Our results suggest that inflammatory cell death, especially in the renal endothelium, contributes to kidney disease. The relatively higher expression of *CASP4*, *CASP11*, and *GBP*s compared to *CASP1*, *PYCARD*, and *NLRP3*, however, may suggest a more prominent role of non-canonical versus canonical inflammasome activation contributing to kidney disease.^49^

### Interplay between IFN and APOL1

The relative expression, localization, and regulation of APOL1 in various renal cell types have been difficult to discern, due in part to the lack of specific antibodies for immunofluorescence analysis.^24,26,45,66^ To address this, we employed a recently developed APOL1 antibody validated for minimal crossreactivity with other APOL family members.^44,45^ Applying this to organoids, we show that IFN-γ rapidly upregulates the expression of APOL1 in tubules, podocytes, and associated endothelial networks. Interestingly, in normal human kidney tissue, APOL1 is detected primarily in glomeruli, with lower expression in tubular cells. This may be due to an uptake of circulating APOL1 rather than differences in cellular expression^25^ or may reflect a low level of circulating IFN *in vivo*, which would be first encountered by the glomerulus through direct exposure to the blood.^44,45^

Although most studies suggest that APOL1-induced podocyte injury is the primary mediator of APOL1-mediated nephropathy,^24,28,29,32,67,68^ there is emerging evidence that APOL1-mediated endothelial injury also contributes to disease progression.^34,35,69^ The link between inflammatory IFN signaling and cardiovascular pathology is well established,^20,70–73^ and disrupted crosstalk between glomerular ECs and podocytes is generally considered to contribute to the progression of kidney disease.^10,74–76^ In this regard, it is interesting that endothelial networks surrounding organoids appeared more susceptible to IFN-γ compared to endothelia within organoids, suggesting possible crosstalk between endothelial and epithelial cells. Similarly, HIV has the highest known increase in odds ratio for developing glomerular disease with an APOL1 high-risk genotype^77^ and is strongly associated with markers of EC injury including thrombotic microangiopathy^78^ and formation of tubuloreticular inclusions.^79^ Studies investigating APOL1 risk variants have suggested various mechanisms explaining cytotoxicity including pathological formation of ion channels, disruption of endo-lysosomal trafficking, or a feedforward loop of inflammasome activation resulting in pyroptotic cell death.^36^ Our results suggest that both ubiquitous inflammatory cell stress and inherent endothelial insult during chronic conditions that upregulate APOL1 expression may contribute synergistically to the etiology of APOL1-mediated nephropathy.

### JAK-STAT inhibition as a therapeutic approach

Because our organoids are generated from human pluripotent stem cells, they express *APOL1* from its endogenous locus, revealing species-specific regulatory pathways, and are readily subjected to controlled conditions. Our organoid model supports the blockade of IFN signaling as a potential therapeutic strategy to mitigate the risk of APOL1-mediated nephropathy. Using the APOL1 signal as a representative and disease-relevant marker, both INCB018424 and baricitinib block IFN-γ-mediated activation of the JAK-STAT pathway. Baricitinib was also shown to reduce the pyroptotic gene signature induced in renal cells and rescue endothelial networks after treatment with IFN-γ. Notably, baricitinib is FDA approved for the treatment of rheumatoid arthritis, alopecia areata, and COVID-19 and thus may also be appropriate to treat kidney-specific inflammatory conditions.^80^ Thus, our study highlights the JAK-STAT signaling pathway as a promising target for simultaneously mitigating cellular inflammatory stress and APOL1 upregulation across renal cell types.

### Study limitations

Much remains unsolved with regard to the effects of IFNs, which are diverse and pleiotropic. We have focused here on IFN-γ, but other IFN subtypes may have unique effects. Additional work is necessary to fully understand the underlying mechanism driving the degradation of endothelial networks we observe in organoid cultures and the physiological relevance of this phenomenon. Importantly, our results *in vitro* only reflect the effect of IFN-γ, whereas the ultimate manifestation in patients will also depend on associated pathological and immunological processes.^81^ Our gene expression and western blot analyses implicate a pyroptotic mechanism; however, the heterogeneous mixture of cells in organoid cultures, and the comparatively small fraction of ECs, raises challenges for demonstrating pyroptosis specifically in ECs. Additional studies with primary ECs, including 3D vascular models,^82^ may provide insight into endothelial-origin-dependent differences. Moreover, the detection of programmed cell death *in vivo* is technically challenging, susceptible to staining artifacts in sections, and often dependent on advanced techniques such as flow cytometry or reporter gene constructs.^83^ Although we demonstrate a causative effect in organoids, we recognize that analyses of patient biopsies are fundamentally correlative and both types of data are complementary in generating disease-relevant hypotheses.

Although organoids are capable of expressing APOL1 in response to IFN, they exhibit altered expression patterns compared to human biopsies. This may reflect an absence of the native biochemical milieu or altered exposure in static culture compared to circulatory exposure physiologically. Further experiments are needed to accurately detect APOL1 in biopsies from patients with inflammatory conditions such as HIVAN to determine whether tubules express APOL1 *in vivo* under these conditions. Unfortunately, such samples are relatively rare and suffer from abundant staining artifacts, which complicates analysis. This study also does not address differences in response to IFN-γ depending on APOL1 genotype. A previous study suggested that IFN-γ stimulation coupled with tunicamycin-induced endoplasmic reticulum stress induces enhanced podocyte dedifferentiation in risk variant APOL1 organoids, compared to a wild-type isogenic control, but only one cell line of each genotype was analyzed.^40^ Further experiments, using a larger cohort of cell lines, would better prove the specific effect of the APOL1 genotype in these cultures.^84^ Cell lines in which *APOL1* can be induced orthogonally from inflammatory stress, for instance using inducible *APOL1* transgenes, would also help assess the potential synergistic effect of endogenous inflammatory and APOL1-induced cell stress toward kidney disease progression. Finally, while we have focused here on the kidneys, it remains unclear whether the phenomenon of vascular injury coinciding with pyroptotic priming might be broadly relevant to other organ systems in which IFN stimulation can have maladaptive consequences.

## STAR★METHODS

Detailed methods are provided in the online version of this paper and include the following:

### RESOURCE AVAILABILITY

#### Lead contact

Further information and requests for resources and reagents should be directed to and will be fulfilled by the lead contact, Benjamin Freedman (benof@uw.edu).

#### Materials availability

PODXL-GFP materials (plasmids & cell lines) are available from the corresponding author upon request, in accordance with institutional material transfer agreements and third party agreements.

#### Data and code availability

- Single-cell RNA-seq data have been deposited at GEO under the accession numbers GSE135663, GSE230848, GSE219185, GSE213030 and are publicly available as of the date of publication. Accession numbers are listed in the key resources table. Original western blot images, microscopy data, and analyzed datasets reported in this paper will be shared by the lead contact upon request.
- Custom code used for quantitative analysis is available as a supplemental word document SI Custom ImageJ scripts and R code used for analysis.
- Any additional information required to reanalyze the data reported in this work paper is available from the lead contact upon request.

### EXPERIMENTAL MODEL AND STUDY PARTICIPANT DETAILS

#### WTC11 iPSCs

This cell line has been deposited at the Coriell Institute for Medical Research under the identifier GM25256, it is derived from a Japanese male donor, age 30–34 at collection. This line was used as the parent line at passage 12 (P12) to generate the GFP-tagged PODXL iPSC cell lines HF22-E8 and HF22-E9 generated in the Freedman and Fu labs at the University of Washington as described in the Method details section.

#### hiPSC GFP-Tubulin

This cell line is available from the Allen Institute with the identifier: AICS-0012:WTC-mEGFP-TUBA1B-cl105 (mono-allelic tag) and was derived from the WTC11 iPSC parent line at passage 33.

#### 1016SevA iPSCs

This cell line was derived from fibroblasts from a male non-African ancestry donor at the Harvard Stem Cell Institute with the identifier RRID:CVCL_UK18.

#### UM77–2 hESCs

This embryonic stem cell line was generated at the University of Michigan and submitted to the NIH with registration no. 0278.

#### iPSC line BXS0114

This cell line is commercially available from ATCC under the identifier ACS-1028 and was derived from the bone marrow of a 31 year old African American Female.

#### iPSC line BYS0110

This cell line is commercially available from ATCC under the identifier ACS-1024 and was derived from the bone marrow of a 33 year old African American male.

#### Primary human adult kidney endothelial cells (AKECs)

AKECs were isolated from normal kidney tissue parts from donors in Ying Zheng’s Lab at the University of Washington under UW IRB #7768 following the protocol described in the method details. Aliquots of AKECs were cryopreserved at passage 2 (P2) in 10% DMSO, 30% FBS and 60% EGM2, thawed as necessary, cultured in AKEC media described in the method details and used up to P5. Available details for donor age, sex, and tissue characteristics are provided:

AKEC35: 74M w/10 × 10 cm R renal mass.

AKEC36: 73M w/11.7 × 7.2 3 5.3 cm L renal mass.

AKEC37: 78F w/7 × 7 × 7.4 cm R renal mass with internal necrosis, Clear Cell Renal Cell Carcinoma.

#### HUVECs

HUVECs from pooled donors were purchased from Lonza (Cat. # CC25–19) (Lot #0000478982, #3001900190, and #0000474578). Aliquots of HUVECs were cryopreserved at P2 in 10% DMSO, 30% FBS and 60% EGM2, thawed as necessary, and cultured in EGM2 (Lonza) until plating for experiments and used up to P7.

### METHOD DETAILS

#### Stem cell culture

Stem cell lines used for kidney organoid differentiations included WTC11 iPSCs and gene-edited 1016SevA iPSCs.^40^ Stem cell lines were cryopreserved in FreSR-S (STEMCELL Technologies) until use, after thawing they were maintained feeder-free in 6-well tissue-culture treated dishes (Falcon) at 37° on 0.5% Geltrex (Gibco) in 2mL mTeSR1 (Stem Cell Technologies) supplemented with 1% penicillin/streptomycin (Gibco) and passaged using ReLeSR (STEMCELL Technologies). UM77–2 hESCs (NIH registration no. 0278) were maintained on 0.5% hESC-qualifi Matrigel (Thermo Fisher) in mTeSR1 and passaged with Versene (Thermo Fisher) in accordance with University of Michigan oversight. All experiments were performed with stem cell lines at passage < P100.

#### Kidney organoid differentiation

Stem cells were differentiated into kidney organoids following an adaptation of our previously published protocol.^38,50^ Briefly, stem cells were dissociated with Accutase (STEMCELL Technologies) and plated at a density of 1–2k cells/cm^2^ into 96-well (Greiner Bio-One) or 24-well plates (Falcon) precoated with 0.5% GelTrex in mTeSR1 supplemented with 10 μM Y-27632 ROCK Inhibitor (STEMCELL Technologies). The following day, scattered, isolated spheroid colonies were sandwiched with another layer of 1.5% GelTrex in mTeSR1 media. Two days after sandwiching, spheroids were treated with 12 μM CHIR (Stemgent) in aRPMI (Thermo Fisher Scientific) for 40–42 h, then changed to RB (aRPMI + 1X Glutamax + 1X B27 Supplement, all from Thermo Fisher Scientific) and replaced every 3 days thereafter. To improve differentiation efficiencies of UM77–2 hESCs and 1016SevA iPSCs, 10 ng/mL noggin (STEMCELL Technologies) was included during CHIR treatment. Additional modifications to the differentiation protocol for 1016SevA iPSCs included CHIR treatment in RB opposed to aRPMI, and addition of the TGF-β1 receptor inhibitor SB-431542 (5 μM, Cayman Chemical) for 4 days immediately following CHIR treatment.

#### Generation of PODXL-GFP iPS cells

Freshly plated, WTC11 iPS cells (Coriell GM25256) at 75% confluence in one well of a 6-well plate were transfected using Lipofectamine Stem (Thermo Fisher) with 1.5 mg each of three plasmids separately encoding Cas9-GFP, a guide RNA targeting the C terminus of human *PODXL* (CCACCGGCAGACCGGACTAG), and a knock-in template gBlock in Bluescript II SK(+) vector (Biomatik) encoding the following GFP sequence:

GGCTCCGGCATGGTGTCCAAGGGCGAGGAGCTGTTCACCGGGGTGGTGCCCATCCTGGTCGAGCTGGACGGCGACGTAAACGGCCACAAGTTCTCCGTGCGGGGCGAGGGCGAGGGCGATGCCACCAACGGCAAGCTGACCCTGAAGTTCATCAGCACCACCGGCAAGCTGCCCGTGCCCTGGCCCACCCTCGTGACCACCCTGACCTACGGCGTGCAGAGCTTCTCCCGCTACCCCGACCACATGAAGCGCCACGACTTCTTCAAGAGCGCCATGCCCGAAGGCTACGTCCAGGAGCGCACCATCTCCTTCAAGGACGACGGCACCTACAAGACCCGCGCCGAGGTGAAGTTCGAGGGCGACACCCTGGTGAACCGCATCGAGCTGAAGGGCATCGACTTCAAGGAGGACGGCAACATCCTGGGGCACAAGCTGGAGTACAACTTCAACTCCCACAACGTCTATATCACCGCCGACAAGCAGAAGAACGGCATCAAGGCCAACTTCAAGATCCGCCACAACGTGGAGGACGGCTCCGTGCAGCTCGCCGACCACTACCAGCAGAACACCCCCATCGGCGACGGCCCCGTGCTGCTGCCCGACAACCACTACCTGTCCACCCAGTCCAAGCTGTCCAAAGACCCCAACGAGAAGCGCGATCACATGGTCCTTCTGGAATTCGTGACCGCCGCCGGGATCACTCACGGCATGGACGAGCTGTACAAGGGCAGCGGCCATCACCATCACCATCACGCGTAG,

where the final TAG is the *PODXL* stop codon. 72 h after transfection, the cells were dissociated and sorted to purify GFP^+^ cells (~5% of cells) using a FACS Aria cell sorter. Purified cells were collected in mTeSR1 + 10 μM ROCK inhibitor, pelleted, and replated at densities of 2,500, 5,000, or 10,000 cells/well in cloning media consisting of mTeSR1 + 10% CloneR (STEMCELL Technologies) in a 6-well plate coated with 1% GelTrex (Thermo Fisher). Two days later, the media was changed to fresh cloning media. The following day, 500 μL of cloning media was added to each well. The following day, the media was changed to mTeSR1 and maintained thereafter with daily media changes until small colonies had formed. Colonies were manually microdissected and each was transferred into one well of a 96 well plate. The 96-well was then split into duplicate plates which were grown to 80% confluence. One plate was used for DNA purification and one plate was cryopreserved for future thawing. After confirming proper insertion with primers specific to the insert and genomic DNA, individual colonies were thawed and expanded into new iPS cell lines. Additional details on cell line generation and validation are available in Figure S2.

#### Recovery of human kidney tissue and isolation of primary adult kidney endothelial cells

Adult human kidney tissue was obtained from normal tissue parts of the renal cortex of nephrectomies performed for renal masses or transitional cell carcinoma, and used to isolate primary adult kidney microvascular endothelial cells (AKECs) under UW IRB # 7768 as previously described.^85^ Briefly, the fresh kidney tissue was finely cut in a 100 mm Petri dish using a razor blade in 1 mL of cold Dulbecco’s modified Eagle’s medium (DMEM/F12 Medium, GIBCO) containing 1% penicillin-streptomycin (PS, GIBCO), 0.2 mg/mL Liberase DL (Roche Applied Science) and 100 U/mL DNase I (Roche Applied Science) then transferred in 50 mL tubes and incubated at 37°C for 30 min in a shaking water bath. The enzymatic digestion was inactivated by adding 2.5 mL of DMEM/F12 medium containing 10% fetal bovine serum (FBS, GIBCO). The single cell suspension was passed two times through a 40 μm cell strainer (Fisher) to remove glomeruli and multicellular debris. Cells were then centrifuged at 1300 rpm at 4 C for 10 min, washed once in isotonic phosphate buffered solution (PBS) and resuspended in 0.5 mL of MACS buffer (Miltenyi Biotec). The single cell suspension was then depleted of epithelial cells using magnetic beads conjugated with antibodies against the epithelial cell marker CD326 (Miltenyi Biotec). Epithelial depletion was performed according to the manufacturer’s instructions (Miltenyi Biotec). After epithelial depletion, remaining cells were cultured in T75 flasks coated with 0.2% gelatin (Sigma) for five days in “AKEC medium” comprised of basal EBM-2 (GIBCO) containing 1% antibiotic-antimycotic (Life Technologies), 10% FBS, 100 g/mL endothelial cell growth supplements (ECGS) (Sigma), 50 μg/mL Heparin (Sigma), and 40 ng/mL VEGF-A (R&D). The culture was within a humidified incubator at 37°C supplied with 5% O2 and 5% CO2. To purify and sort the AKECs, cell mixtures grown in T75 fl were detached using 0.05% trypsin/EDTA, centrifuged and washed once in cold PBS. Cell pellets were then resuspended in FACS buffer (1% BSA, 0.5 mM EDTA (pH7.4) in PBS) and incubated with Fc blocking reagent (Biolegend) for 10 min on ice. Directly conjugated anti-CD45 (Biolegend), and PECAM (BD Pharmingen, clone WM59) were added to the cell mixture at 1:100 dilutions and incubated for 20 min on ice. After incubation, cells were washed with ice-cold FACS buffer twice, followed by FACS sorting using BD FACS Aria II (BD Biosciences). After sorting, the purified CD45^−^, CD31^+^ cells were immediately cultured in AKEC medium for up to five passages.

#### Agonist and inhibitor treatment in organoids and endothelial monocultures

Mature organoids and endothelial cell monocultures were treated with a variety of inflammatory agonists and pharmacological compounds with concentrations and timing indicated alongside the relevant results. All pharmacological compounds were delivered in 0.1% DMSO (Corning) vehicle in RB for organoids or AKEC medium for endothelial experiments. All control and IFN-γ treated conditions were also treated with 0.1% DMSO for consistency. All reagents were reconstituted per the manufacturers’ instructions and stored at −20°C in single use aliquots to avoid freeze-thaw. Details on sourcing and concentrations used for all reagents are available in the STAR Methods key resource table (KRT). The specific concentrations of pharmacological inhibitors used in organoid assays were determined as the maximum concentration without observable cytotoxicity in iPSCs using a CellTiter-Glo assay (Promega), and compared to previously established values.^24,46^

#### Kidney organoid and normal human kidney preparation and cryosectioning

This study was approved by the University of Washington institutional review board (STUDY00001626). Preimplantation donor kidney biopsies (*n* = 3) were stabilized in Michel’s medium and then frozen in optimal cutting temperature compound (OCT) for clinical testing. Residual frozen tissue was used to prepare ~7 μm cryosections and stored at −80°C then fixed in 4% paraformaldehyde (PFA, Electron Microscopy Sciences)/PBS (Thermo Fisher Scientific) for 15 min immediately prior to immunofluorescent staining.

Organoids for cryosectioning and immunocytochemistry were hand-picked following the indicated treatment to detach them from the well plate, fixed in 4% PFA, infiltrated with a sequential gradient of sucrose in PBS, and embedded in 20% sucrose/OCT (Tissue-Plus, Thermo Fisher Scientific) as previously described.^50^ Embedded organoids were cryosectioned at 5 mm thickness, mounted on superfrost slides (Fisherbrand) and stored at −80° C until staining.

#### Immunofluorescence and live time-course microscopy

Adherent organoid cultures were fixed in 1:1 ratio of culture media and 8% PFA (4% working solution) for 15 min at room temperature. After fixing, samples were immediately washed in PBS x3 and stored at 4°C until staining. Cryosections of organoids and kidney tissue were rehydrated and washed with PBS prior to staining. Adherent cultures and cryosections were both blocked in 5% donkey serum (Millipore)/0.3% Triton X-100/PBS for 1 h at room temperature and incubated overnight in 3% BSA (Millipore)/PBS/CaCl_2_ (100 μM) (AbDil) with primary antibodies. Primary solution was washed x3 with PBS then incubated in AbDil with Alexa-Fluor secondary antibodies (Invitrogen) and 4′,6′ -diamidino-2-phenylindole (DAPI, 10 μg/mL, Invitrogen) overnight then washed x3 in PBS and protected from light for storage. Details on antibodies used including sourcing and dilutions are available in the KRT. High resolution representative z-slices were acquired using an inverted Nikon A1R confocal microscope equipped with a 40 × air objective. For quantitative image analyses, images were acquired using an Olympus IX83 microscope equipped with a disk spinning unit (DSU, Evident) and cellSense software. To ensure the full thickness of the organoid was analyzed for APOL1 and GSDMD fluorescent signal intensity quantification, 100 μm thick confocal z-stacks (10 μm/slice) were acquired using the DSU, imaged at 20x. For quantification of endothelial network density, 10 μm thick confocal z-stacks (1 μm/slice) were acquired using the DSU through the thickness of the EC network, imaged at 10x. Images were only acquired for regions of interest (ROIs) containing a single organoid, and the ROI was positioned to capture the entire organoid body and maximal associated endothelial network. For quantification of PODXL-GFP fluorescent signal intensity during time course imaging of live organoids, 100 μm thick confocal z-stacks (10 μm/slice) were acquired using the DSU, imaged at 10x. Wide field imaging on the Olympus IX83 was used to quantify endothelial monoculture nuclear densities (imaged at 10x) and average cellular APOL1 fluorescent intensities (imaged at 20x).

#### Quantitative image analyses

A variety of semi-automated image analyses were developed and performed in ImageJ for quantitative fluorescence microscopy assays. Raw ImageJ scripts written as IJ1 Macros are available as a supplementary methods section with accompanying notes on necessary user edits for adaptation. Accurate quantification of fluorescent images is highly dependent on uniform staining and imaging across conditions, as such, care was taken to ensure that all conditions within a given replicate were identically stained and then all imaging for a set was performed within a 1–2 day span to minimize the chance of signal fading. Quantification of fluorescent signal intensities for each assay is presented as a normalized relative metric after subtraction of the background signal. The background signal for all assays was defined as the average minimum pixel intensity across all images within a given set. For all analyses, Z-stacks were collapsed and quantification was performed on their maximum intensity projections.

Average APOL1 and GSDMD fluorescent signal within an organoid body was determined by first manually tracing the body of an organoid using LTL and PODXL staining to define its perimeter (Figures S7A and S7B). The average raw pixel intensity for the channel of interest within the manual trace was then measured using ImageJ and normalized to the control condition.

Relative changes in PODXL-GFP signal over time were quantified by sequential imaging of particular organoids across conditions. Bright field images were used to determine and manually trace the perimeter of organoids on each day. The area and the integrated GFP signal intensity within the trace for each organoid was measured on each day using ImageJ and normalized to its initial measured value.

Quantification of CD31^+^ network density associated with an organoid, and APOL1 signal intensity within the EC network was measured using a custom ImageJ script provided in the supplemental methods. Briefly, the outline of the organoid body was manually traced within an ROI based on PODXL, DAPI and brightfield images to delineate EC networks occurring within the organoid versus in the surrounding stroma (termed stromal vessels). A threshold intensity for a given replicate was then subjectively determined to generate CD31^+^ binaries as masks. The APOL1 fluorescent intensity was then measured within CD31^+^ binary areas outside of the organoid body. Quantification of APOL1 fluorescent intensity was restricted to stromal vessels; network areas measuring less than 2000 μm^2^ were excluded as spurious noise.

Endothelial monoculture nuclear densities were quantified using particle counting in ImageJ. To account for differences in cell density depending on time and treatment, a custom ImageJ script was used to determine the average APOL1 signal intensity within cell bodies. DAPI staining was used to approximate the location of the cell body to generate a binary as a mask surrounding the nucleus (Figure S7C). The average fluorescent intensity of APOL1 within the binary masks were then calculated. All conditions and replicates across lots and donors for both HUVECs and AKECs were stained in parallel to allow comparison between cell lines and normalized to the HUVEC pretreatment condition. The average value across 4 regions of interest within a culture well was used as the single reported metric per lot or donor. CD31^+^ area was determined by uniformly thresholding images to generate binaries of junctional CD31, but excluding intracellular CD31 which stained more faintly.

#### Western blot analysis

Organoid cultures were lysed in RIPA buffer containing protease and phosphatase inhibitors. Protein concentrations were determined using the Pierce BCA protein assay (Thermo Scientific) with bovine serum albumin (BSA) as a standard. Protein samples were denatured in Laemmli sample buffer at 95°C for 5 min. Denatured proteins were separated on 4–15% polyacrylamide gels using sodium dodecyl sulfate-polyacrylamide gel electrophoresis (SDS-PAGE) at 100 V. Precision Plus Protein Kaleidoscope Prestained Protein Standards (Bio-Rad) were used as molecular weight markers. Proteins were transferred onto low fluorescence polyvinylidene difluoride (PVDF) membranes at 80 V for 2 h in transfer buffer containing 10% methanol. Membranes were blocked in 5% non-fat milk in Tris-buffered saline with 0.1% Tween 20 (TBST) for 1 h at room temperature. Membranes were incubated with the appropriate primary antibody in blocking solution overnight at 4°C. After washing with 4x TBST, membranes were incubated with an appropriate species-specific, fluorescence or HRP-conjugated secondary antibody overnight at 4°C. Membranes were again washed, then signals were detected using a ChemiDoc MP Imaging System (BioRad, for fluorescence), or by exposing the blot to ECL Blotting film (Prometheus 30–810L) processed in a darkroom film processor. The blots were then reprobed for additional primary and secondary antibodies of different molecular weights, repeating the above procedure. Experiments were replicated from 4 independent differentiations to ensure reproducibility of results. Immunoblots were quantified using the ImageJ Gel Analyzer. Protein bands were normalized to the loading control (GAPDH) for each lane.

#### Organoid culture for APOL1 ELISA and single cell RNA-seq

Kidney organoids generated from UM77–2 human embryonic stem cells (NIH approval #: NIHhESC-14–0278) were treated on D23 of cell culture. Organoids were harvested after 24 h treatment with with 25 ng/ml of IFN-γ (R&D Systems) resuspended in sterile deionized water and/or 1000nM of baricitinib (MedChemExpress HY-15315, in DMSO) added 1 h prior to IFN-γ. For ELISA analysis, organoids were washed in ice-cold PBS and scraped into Cell Lysis Buffer 2 (R&D Systems) supplemented with Halt Protease and Phosphatase inhibitor Cocktail (Thermo). 2–3 wells were combined for each biological sample to provide adequate protein for analysis and *n* = 3 independent experiments. APOL1 was measured using the Human APOL1 ELISA kit (ProteinTech). Samples were diluted 2 to 4-fold and processed in (technical) duplicate following the manufacturer’s protocol. Absorbance was measured with a VersaMax ELISA plate reader, and results were calculated with SoftMax Pro (Molecular Devices). Values were normalized to total protein content as assessed by Pierce BCA protein assay (Thermo Scientific).

For scRNA-seq, whole wells were harvested and dissociated into single cells on ice using cold active protease solution [5 μM CaCl2, 10 mg/mL *Bacillus licheniformis* protease (Sigma, P5380) and 20 U/ml DNAse (Qiagen, 79254) in DPBS (Gibco, 14190144)]; dissociation was halted with 10% fetal bovine serum (Gibco, A3160501) in ice-cold PBS. Single cell RNA library generation and sequencing was performed on a 10x Genomics Chromium platform at the University of Michigan’s Advanced Genomics Core.

#### Oxygen consumption rate of iPSC-derived endothelial cells and analysis with seahorse analyzer

Endothelial-like cells were differentiated from iPSC lines BXS0114 (ATCC) and BYS0110 (ATCC) using a monolayer approach, as previously described.^86^ Briefly, WNT signaling was activated to generate mesoderm cells for 4 days, and cells underwent endothelial specification with VEGF and FGF. On day 10, cells were sorted with CD144 magnetic beads using MACS magnetic sorting.

Oxygen consumption rate (OCR) analysis was performed with a Seahorse XF96 analyzer according to the manufacturer’s recommendations (Agilent Technologies). Briefly, cells were seeded in a Seahorse 96-well plate (Agilent Technologies) and incubated overnight at 37°C in a CO2 incubator. They were then treated with vehicle, 5 ng, 10 ng, 25 ng, or 50 ng of IFN-γ for 24 h. After measurement of basal OCR, Oligomycin (3 μM), FCCP (1 μM) and AA/R (1.5 μM/3 μM) were sequentially added to the cells, and the OCR was monitored over time according to the manufacturer’s recommendation.

#### Single-cell transcriptomic analyses

Organoid scRNA-seq data processing was performed using Seurat v4.0^68^; cells expressing >500 genes were included in the analysis. The processing steps include log transformation, scaling or linear transformation using default settings, highly variable gene identification, dimensionality reduction using principal component analysis (PCA) and Uniform Manifold Approximation and Projection (UMAP), batch correction using harmony function embedded in Seurat and unsupervised clustering at 1.0 resolution. The R Data Serialization (RDS) object was uploaded for visualization (dot plot) and analysis to CZ CELLxGENE (Chan Zuckerberg Initiative, https://cellxgene.cziscience.com/). Data are available at the National Center for Biotechnology Information’s Gene Expression Omnibus (GEO), accession number GSE230848.

Because an endothelial cell signature was not resolvable in our newly acquired scRNA-seq dataset we re-analyzed our previously published data investigating IFN-γ treatment in the 1016SevA cell line.^40^ The Seurat (V4.1.1) R package was used for scRNA-seq analysis of the Liu et al. data (GSE135663) downloaded from the NIH GEO repository. The sample key from the GEO NIH website utilized *N* = 4 G0 controls, *N* = 4 G1 controls, *N* = 2 G0 IFN exposure, *N* = 2 G0 IFN+Tun, *N* = 2 G1 IFN and *N* = 2 G1 IFN+Tun conditions in cell clustering. Data within each condition was subset for nFeature_RNA >300, nFeature_RNA <4000 and percent.mt < 10 and normalized with nfeatures = 2000. The conditions were integrated using the first 20 dimensions. Data was scaled with the ScaleData() function followed by RunPCA() with 30 PCs and RunUMAP() was used for clustering at a resolution of 0.2. Cell identities were asigned as reported in the Lin et al. publication. Data were visualized using the DotPlot() function with selected genes and cell types with the G0 genotype.

#### Gene ontology pathway analysis

Using the Liu et al. kidney organoid single cell data^40^ we conducted a Gene Ontology analysis in the APOL1 G0 genotype cells between the interferon-gamma stimulation and the G0 control cells. We first found the differentially expressed genes by using Seurat’s FindMarkers() function and a multiple testing adjusted *p*-value<0.05. We then selected the genes that are upregulated in the interferon-gamma cells. Using the clusterProfi er R packages we then used the enrichGO() function with the org.Hs. e.g.,.db gene database with the SYMBOL keys and the Biological Process (BP) Gene Ontology. We plotted the first 20 most significant GO terms and provided a supplemental csv file of all significant GO terms tested for enrichment for each cluster of cells (Table S1).

Details on patient demographics, clinical outcomes, sample collection, and Gene Ontology (GO) analysis from the COVID-19 Host Response and Clinical Outcomes (CHROME, UW IRB: 9763 and 6878) study are previously published.^42^ In brief, subjects were eligible if they were admitted to an ICU for symptoms suggestive of SARS-CoV-2 infection. Infection was defined by a positive RT-PCR for SARS-CoV-2 from a nasopharyngeal swab. Urine samples were collected within 24 hs of ICU admission and proteomic profiling was performed using the SomaScan Platform (Somalogic) that contains 5284 SOMAmer aptamers that bind to protein analytes. The generalized Berk-Jones (GBJ) test was performed on the mean normalized relative fluorescence values for protein-aptamer sets corresponding to a GO pathway. GO term analysis was pared down to 186 non-redundant GO terms with a false discovery rate (FDR) < 0.05 (Table S2). Terms related to IFN signaling or cell death responses were plotted with R Software (R Core Team [2023]).

#### Clinical differential gene expression analysis and Ingenuity Pathway Analysis

Differential gene expression analysis on bulk RNA-seq and snRNA-seq from patient biopsies were based on a previously published study available through NEPTUNE (NCT01209000). NEPTUNE is a prospective study of patients with proteinuria, recruited from 21 sites at the time of their first clinically indicated kidney biopsy. Additional details on participant demographics, clinical characteristics, and unbiased clustering based on biopsy molecular signatures is previously published.^53^ Unfiltered voom-transformed normalized reads from bulk RNA-seq available through GEO (GSE219185) were analyzed with the R Software (R Core Team [2023]) linear models for microarray data (limma) package. Patient cluster designations were provided by NEPTUNE. Details for snRNA-seq including sample preparation and nuclear cluster annotation were previously published,^53^ and data is available through GEO (GSE213030). Dot plots for snRNA-seq were generated analogous to organoid scRNA-seq data using CZ CELLxGENE filtering for the endothelial cluster only. Upstream regulator analysis in Ingenuity Pathway Analysis (IPA; Qiagen) was performed on differentially expressed genes in the “severe” cluster (absolute fold change >1.5, adjusted *p*-value <0.05) observed in bulk RNA-seq to predict upstream regulation of IFN-γ on pyroptosis-associated genes.

#### GeoMx digital spatial profiling and paired IHC assessment

Spatial transcriptomic data using the nanoString Cancer Transcriptome Atlas probeset (~1,852 genes) was generated in a previously published study using the nanoString GeoMx Digital Spatial Profi er.^56^ The data consists of gene expression at the resolution of the individual glomeruli obtained from *n* = 3 HIV patients and *n* = 3 COVID patients with collapsing glomerulopathy. Glomeruli from *n* = 3 histologically normal kidney biopsies were used for comparison. Since not all glomeruli within a patient’s biopsy may have collapsing histology, for the experiment, each glomerulus in the experiment was annotated as having collapsing or normal histology, in addition to the patient’s known disease status. The expression levels of CD31 and CD34 from *n* = 7 collapsing glomeruli from these HIV and COVID patients was compared to *n* = 12 normal glomeruli. Immunohistochemistry for CD31 (clone JC/70A, Invitrogen) with counterstaining for hematoxylin was performed on a Leica Bond autostainer per protocols established in the clinical laboratory at the University of Washington Medical Center, Seattle, Washington, USA.

### QUANTIFICATION AND STATISTICAL ANALYSIS

Statistical analysis was performed using GraphPAD Prism (La Jolla, CA). For all organoid experiments, *n* equals the total number of organoids pooled across at least 3 independent experimental datasets. For endothelial monoculture experiments *n* = 3 HUVEC lots and 3 AKEC donors. Data are either presented as bar plots with mean ± S.D. and all *n* plotted, or as violin plots with median and quartiles indicated. Data were analyzed using t-tests, two-way ANOVA, or one-way ANOVA with Tukey post-hoc testing for multiple comparisons and are indicated for each graph. All significant differences are indicated, but comparisons without significant differences are not indicated unless notable.

## Supplementary Material

Table S2. Non-redundant Gene Ontology terms from CHROME cohort showing upregulated pathways in admitted patients with COVID-19 infection compared to admitted patients testing negative for COVID-19, related to Figure 7

Table S1. Gene Ontology analysis from Liu et al.’s organoid scRNA-seq showing upregulated pathways for all cell types with IFN-γ treatment, related to Figures 5 and S4

Document S1. Figures S1‒S7

## Figures and Tables

**KEY RESOURCES TABLE:** 

REAGENT or RESOURCE	SOURCE	IDENTIFIER

Antibodies		

ApolipoproteinL-1 (APOL1) mouse monoclonal IgG (IF ~8 mg/mL)	Genentech	4.17A5
ApolipoproteinL-1 (APOL1) rabbit monoclonal IgG (IF ~8 mg/mL)	Genentech	5.17D12
Gasdermin-D (GSDMD) rabbit polyclonal IgG (IF 1:100)	Proteintech	Cat# 20770-1-AP; RRID:AB_10696319
Gasdermin-D (GSDMD) rabbit polyclonal IgG (WB 1:500)	ThermoFisher	Cat# PA5-115330; RRID:AB_2899966
Podocalyxin (PODXL) Goat polyclonal IgG (IF 1:500)	R&D Systems	Cat# AF1658; RRID:AB_354920
Lotus Tetragonolobus Lectin (LTL), Biotinylated (IF 1:500)	Vector Labs	Cat# B-1325-2
CD31-PE, recombinant IgG (IF 1:100)	Miltenyi Biotec	Cat# 130-117-225; RRID:AB_2727880
Synaptopodin (SYNPO) Goat polyclonal IgG (IF 1:300)	Santa Cruz	Cat# sc-21537; RRID:AB_2201166
Zonula Occludens-1 (ZO-1) rabbit polyclonal IgG (IF 1:300)	Invitrogen	Cat# 61-7300; RRID:AB_138452
Plasmalemmal vesicle-associated protein (PV1) Mouse monoclonal IgG (IF 1:500)	abcam	Cat# ab81719; RRID:AB_1658370
GFP chicken polyclonal IgY (WB 1:1000)	Invitrogen	Cat# A10262; RRID:AB_2534023
β-Actin (13E5) Rabbit mAb (WB 1:1000)	Cell Signaling	Cat# 4970; RRID:AB_2223172
OCT-4 rabbit polyclonal IgG (IF 1:500)	Abcam	Cat# ab19857; RRID:AB_445175
Acetylated α-tubulin (Act-tub) monoclonal IgG (IF 1:500)	Sigma	Cat# T7451; RRID:AB_609894
GAPDH Rabbit mAb (WB 1:2000)	Cell Signaling	Cat# 2118; RRID:AB_561053
Stat1 (D1K9Y) Rabbit mAb (WB 1:1000)	Cell signaling	Cat# 14994; RRID:AB_2737027
CD31 Monoclonal Antibody (JC/70A)	Invitrogen	Cat# MA5-13188; RRID:AB_10982120
ImmPress anti-mouse reagent	Vector Laboratories	Cat# MPX-2402-15

Chemicals, peptides, and recombinant proteins		

Baricitinib (DMSO, 1 μM)	MedChemExpress	HY-15315
BX795 (DMSO, 1 μM)	MedChemExpress	HY-10514
INCB018424 (DMSO, 20 μM)	Selleckchem	S1378
SB203580 (DMSO, 15 μM)	Selleckchem	S1076
WHI-P131 (DMSO, 20 μM)	BioVision	1853
SP600125 (DMSO, 20 μM)	BioVision	1669
PD98059 (DMSO, 25 μM)	STEMCELL Tech	72172
Bay 11-7085 (DMSO, 1 μM)	MedChemExpress	HY-10257
TG101348 (DMSO, 1 μM)	BioVision	2314–1
Tunicamycin (DMSO, 1 μM)	Tocris	3516
Pan Caspase Inhibitor Z-VAD-FMK (DMSO, 30 μM)	rndsystems	FMK001
Recombinant Human interferon-γ (IFN-γ, H2O and 0.1% BSA, 100 ng/mL unless otherwise noted)	Peprotech	300–02
Lipopolysaccharide (LPS, H2O, 1 μg/mL)	List Biological Labs	product #434
Recombinant Flagellin (H2O, 1 μg/mL)	PROSPEC	PRO-1240
High molecular weight Polyinosinic- polycytidylic (poly(I:C), H2O, 20 μg/mL)	Invivogen	tlrl-pic-5
SB-431542 (DMSO, 5 μM)	Cayman Chemical	S1067

Critical commercial assays		

Seahorse XF Cell Mito Stress Test Kit	Agilent Technologies	103015–100
Seahorse XFe96 Cell Culture Microplates	Agilent Technologies	103794–100
Seahorse XFe96/XF Pro sensor cartridge	Agilent Technologies	W26923

Deposited data		

Liu et al. scRNAseq	NIH GEO database	GEO: GSE135663
Organoid scRNAseq	NIH GEO database	GEO: GSE230848
NEPTUNE bulk RNAseq	NIH GEO database	GEO: GSE219185
NEPTUNE snRNAseq	NIH GEO database	GEO: GSE213030

Experimental models: Cell lines		

WTC11 iPSCs derived from a Japanese male donor	Coriell	GM25256
WTC11 iPSCs PODXL-GFP	B Freedman & H Fu Labs, University of Washington	HF22-E8
WTC11 iPSCs PODXL-GFP	B Freedman & H Fu Labs, University of Washington	HF22-E9
GFP-Tubulin	Allen Institute/Coriell	AICS-0012
1016SevA iPSCs derived from fibroblasts from a non-African ancestry donor	Harvard Stem Cell Institute	N/A
UM77-2 hESCs	J Harder Lab, University of Michigan	NIH registration no. 0278
iPSC line BXS0114 (African American Female)	ATCC	ACS-1028
iPSC line BYS0110 (African American Male)	ATCC	ACS-1024
Primary human adult kidney endothelial cells	Y Zheng Lab, University of Washington	AKEC35
Primary human adult kidney endothelial cells	Y Zheng Lab, University of Washington	AKEC36
Primary human adult kidney endothelial cells	Y Zheng Lab, University of Washington	AKEC37
HUVECs #1 (pooled donors, Lot #0000478982)	Lonza	CC-2519
HUVECs #2 (pooled donors, Lot #3001900190)	Lonza	CC-2519
HUVECs #3 (pooled donors, Lot #0000474578)	Lonza	CC-2519

Oligonucleotides		

CCACCGGCAGACCGGACTAG	IDT	HF22

Recombinant DNA		

pGuide (modified for oligo HF22)	Addgene	64711
pCas9_GFP	Addgene	44719

Software and algorithms		

ImageJ 1.54f	NIH	N/A
R Software (R Core Team [2023])		N/A
Seurat v4.0		N/A
CZ CELLxGENE	Chan Zuckerberg Initiative	N/A
Ingenuity Pathway Analysis (IPA)	Qiagen	N/A

## Highlights

- IFN-γ exposure degrades endothelial networks in kidney organoids
- IFN-γ induces upregulation of APOL1 and pyroptosis- but not apoptosis-associated genes
- Inhibition of JAK-STAT signaling or pan-caspase rescues endothelial networks
- IFN and pyroptotic genes are upregulated in patient kidneys with accelerated renal failure
