# Supplementary material for: Interferon-γ induces combined pyroptotic angiopathy and APOL1 expression in human kidney disease
Source: Cell Rep. Author manuscript; Available in PMC 2024 Jul 2. (PMC11216883; doi:10.1016/j.celrep.2024.114310)
Supplement: Document S1. Figures S1‒S7 [file NIHMS2002293-supplement-Document_S1__Figures_S1_S7.pdf]

Cell Reports, Volume *43*

## Supplemental information

# Interferon-$\gamma$ induces combined pyroptotic angiopathy and APOL1 expression in human kidney disease

**Benjamin A. Juliar, Ian B. Stanaway, Fumika Sano, Hongxia Fu, Kelly D. Smith, Shreeram Akilesh, Suzie J. Scales, Jamal El Saghir, Pavan K. Bhatraju, Esther Liu, Johnson Yang, Jennie Lin, Sean Eddy, Matthias Kretzler, Ying Zheng, Jonathan Himmelfarb, Jennifer L. Harder, and Benjamin S. Freedman**

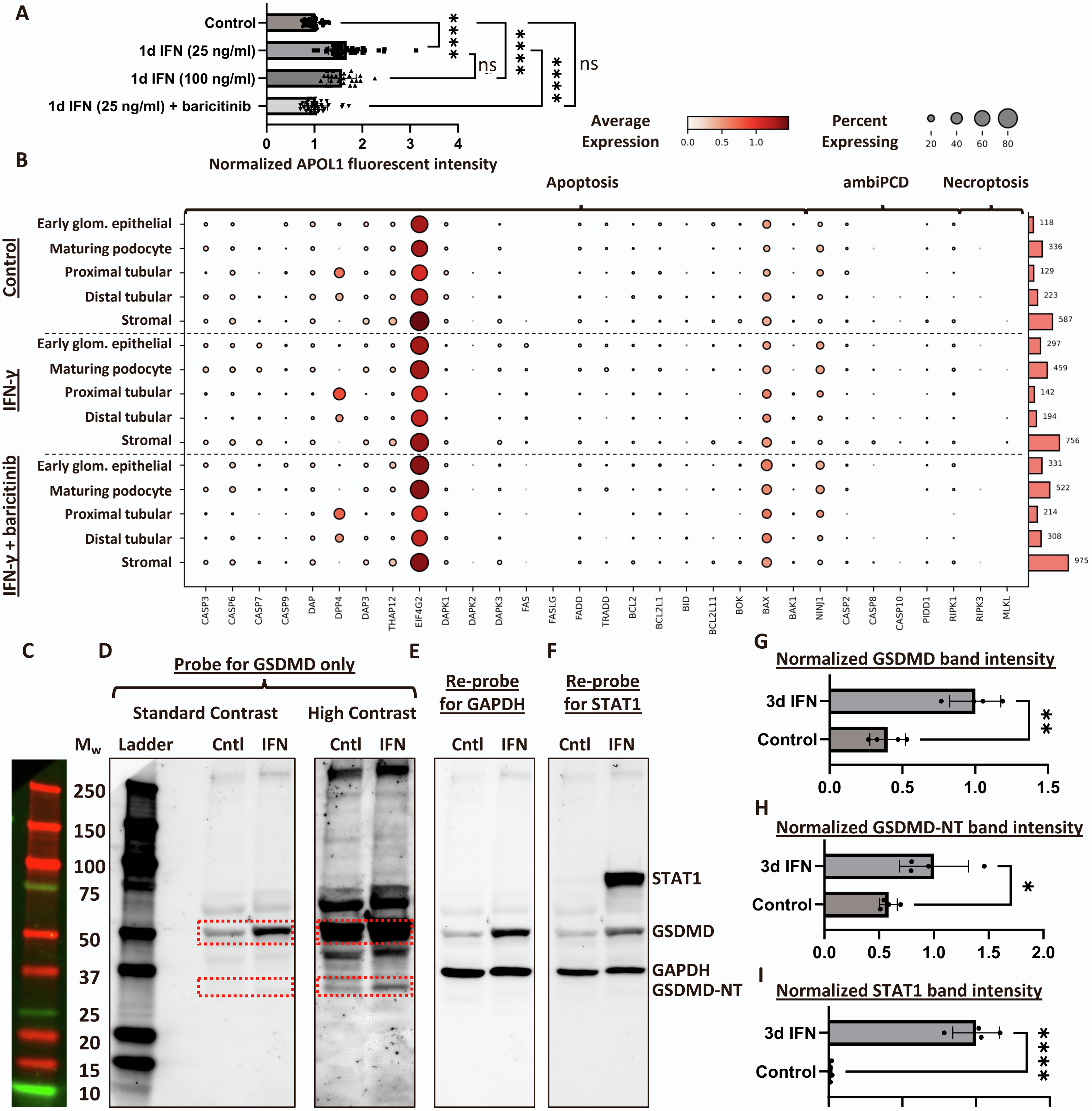


**Figure S1. IFN-γ induces GSDMD protein upregulation and cleavage but does not induce changes in apoptosis-associated gene expression across cell types, Related to Figure 3.** (A) The IFN-γ dose used for scRNA-seq induces comparable upregulation of APOL1 as the standard dose used for morphogenic assays in this study. Average fluorescent intensity of APOL1 throughout organoid quantified with immunofluorescence microscopy at 24 hours (n ≥ 23 organoids pooled across 3 independent experiments) Results are presented as normalized fluorescent intensity (B) Dot plot of apoptosis-, necroptosis-, and ambiPCD- (ambiguous programmed cell death association) associated gene expression in organoids treated with 25 ng/ml IFN-γ for 24 hours ± 1 µM baricitinib. Darker blue dots indicate stronger expression across cells, and dot size reflects percentage of cells in a cluster expressing the indicated gene. Cell counts per type, per condition, are indicated to the right. (C, D, E, F) Representative full-length fluorescent western blot for sequential probing of GSDMD, GAPDH, and then STAT1 for quantification with the (C) ladder imaged across wavelengths to visualize all bands, (D) initial staining for only GSDMD with full length and N-terminal GSDMD fragment boxed in red, (E) subsequent re-probing for GAPDH, and then (F) STAT1. (F, G) Quantification of band intensities for (F) GSDMD and (G) STAT1 corrected for GAPDH band intensity and normalized to treatment condition. (n = 4 independent differentiations). Mean ± S.D. Significance was calculated using one-way ANOVA with Tukey's multiple comparisons test (A) or a two-tailed t-test (G-I). *$p$<0.05 **$p$<0.01 ***$p$<0.001, ****$p$<0.0001

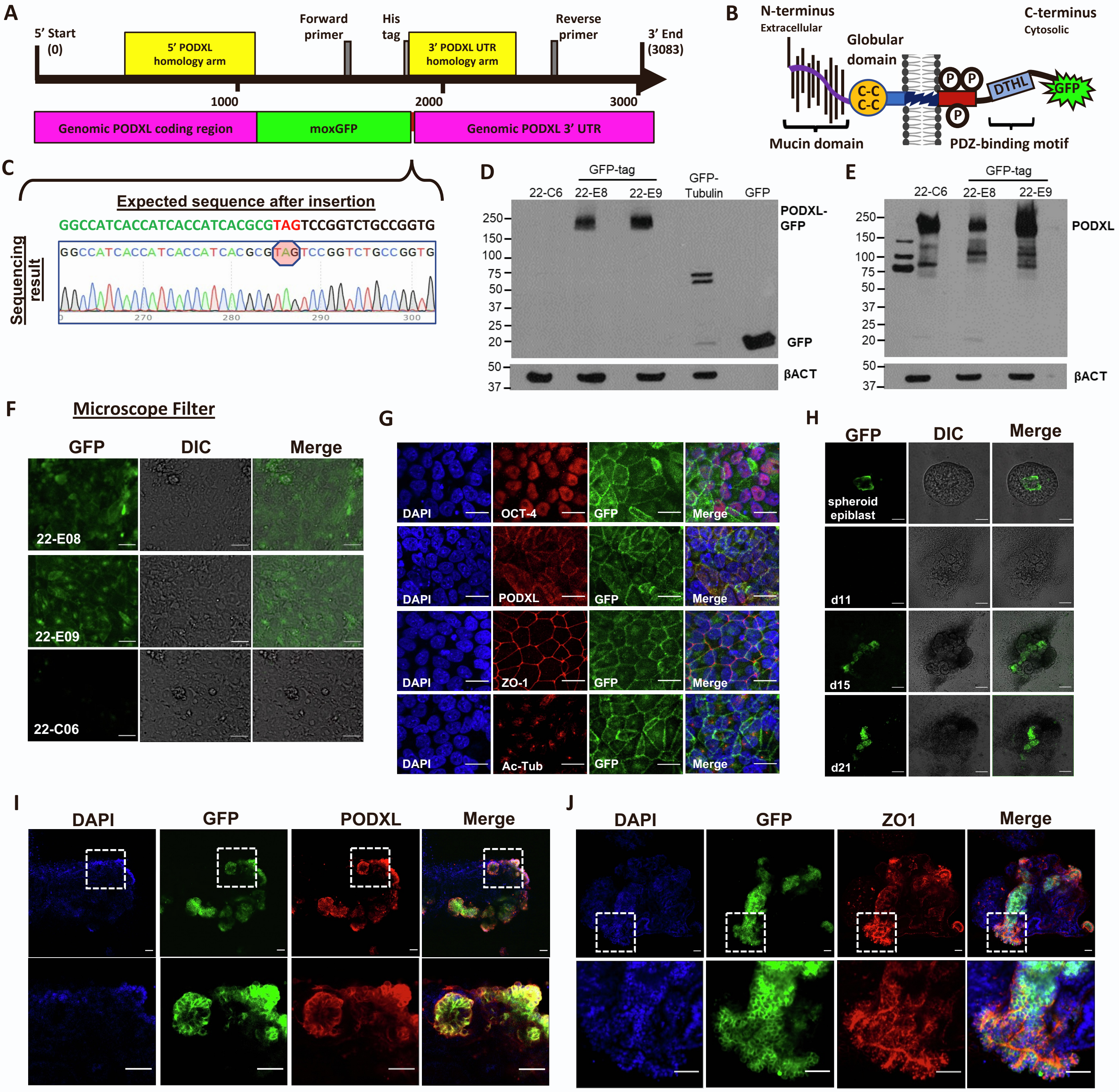


**Figure S2. Generation of PODXL-GFP knock-in iPSC cell lines (22-E08 and 22-E09) and fluorescent microscopy validation of PODXL-GFP expression in iPSCs and organoids, Related to Figure 4.** (A) Summary gene map for moxGFP knock-in and primer locations for sequencing validation. (B) Cartoon schematic of GFP-PODXL protein structure showing location of moxGFP tag. (C) Sequencing result for genomic PODXL in 22-E09 iPSC cell line showing that the expected sequence after insertion matched the sequencing result, confirming proper insert location. (D) Western blot analysis confirming expression of GFP in gene edited 22-E08 and 22-E09 cell lines and in GFP-Tubulin and GFP cell lines (positive controls) but absence in 22-C06 (negative control). (E) Western blot analysis confirming expression of PODXL in each of the newly generated clonal lines. (F) Live fluorescent images of 22-E08, 22-E09 and 22-C06 iPSC cell lines. Scale bars 20 µm. (G) Co-expression of OCT4, PODXL, Act-Tub, and ZO1 with PODXL-GFP in 22-E08 iPSC cell line. Scale bars 40 µm. (H) Live imaging of PODXL-GFP expression in kidney organoids during differentiation. Time course imaging of a representative organoid at the spheroid epiblast state (scale bars 20 µm) and on days 11, 15 and 21 after plating (scale bars 100 µm). (I,J) Immunofluorescent validation of PODXL-GFP expression and proper localization in mature kidney organoids (additional detail for Figure 4B). (I) Proper co-localization of PODXL immunofluorescent staining with PODXL-GFP. (J) Co-expression of ZO1 and PODXL-GFP in podocyte clusters. All scale bars 40 µm. βACT, beta-actin; iPSC, induced pluripotent stem cells; DIC, differential interference contrast; PODXL, podocalyxin; GFP, green fluorescent protein; OCT-4, octamer-binding transcription factor 4; ZO-1, Zonula occludens-1; Ac-Tub, acetylated tubulin.

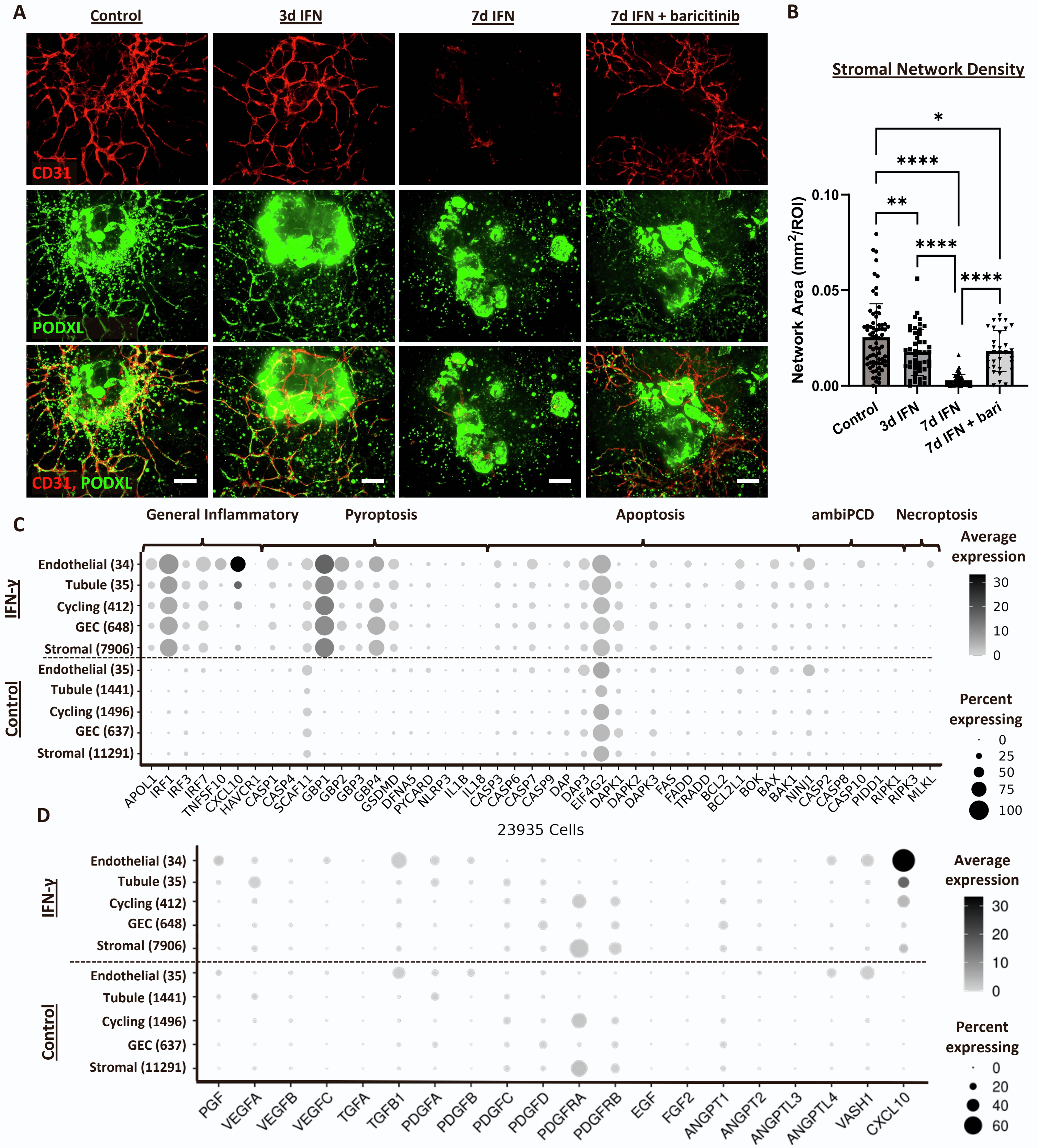


**Figure S3. Confirmation that prolonged IFN-γ induces loss of endothelial networks and that the Liu et al. data set demonstrates changes in pyroptosis- but not apoptosis- or angiogenesis-associated gene expression across cell types, Related to Figure 5.** (A) Automated quantification of CD31+ network density per region of interest (ROI, 0.71 mm$^2$) in stromal area surrounding organoids. (n ≥ 32 organoids pooled between 4 independent experiments). Mean ± S.D. Significance was calculated using one-way ANOVA with Tukey's multiple comparisons test. *$p$<0.05, **$p$<0.01, ****$p$ <0.0001. (B) Representative maximum intensity projections of disk spinning confocal z-stacks restricted to thickness of endothelial networks showing co-localization of CD31 and PODXL. PODXL$^+$ podocyte tufts intentionally oversaturated to show fainter PODXL staining in endothelial networks. Scale bars 100 µm (C) Dot plot of general inflammatory, pyroptosis-, apoptosis-, ambiPCD- (ambiguous programmed cell death association), and necroptosis-associated gene expression and (D) angiogenesis-associated gene expression across organoid cell types treated with 25 ng/ml IFN-γ for 24 hours ± 1 µM baricitinib. Glomerular epithelial cell (GEC). Darker dots indicate increased average expression, and dot size reflects percentage of cells in a cluster expressing the indicated gene. Cell counts per type, per condition, are indicated in parenthesis.

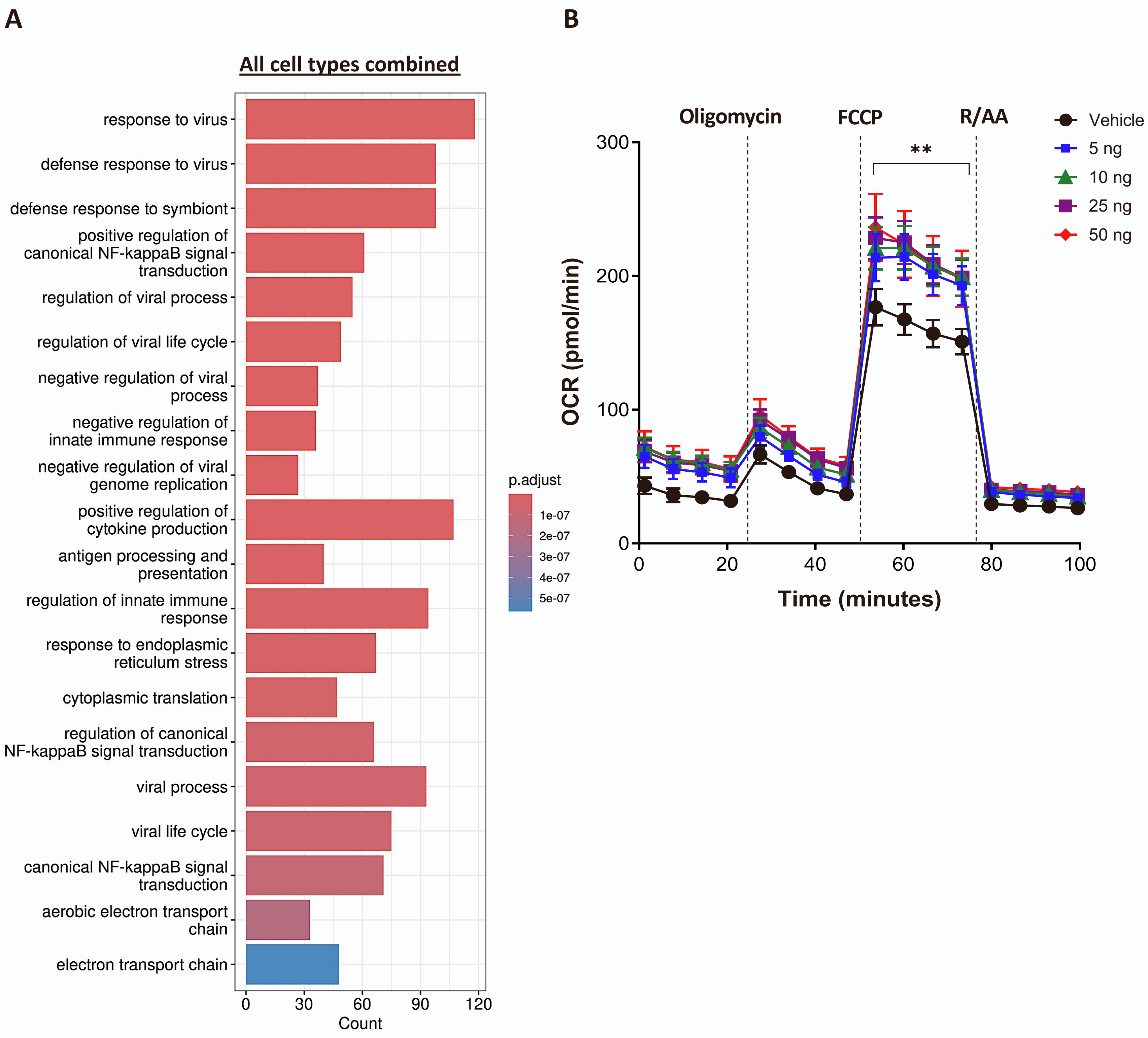


**Figure S4. Changes in mitochondrial respiration are observed with IFN treatment, Related to Figure 5 and Table S1.** (A) Gene Ontology (GO) pathway analysis of scRNAseq results for all organoid cell types pooled comparing 24hr IFN treatment to control from the Liu et al. data set. The top 20 GO terms are shown with their significance (adjusted p-value) and gene count. (B) Changes in oxygen consumption rate (OCR) in iPSC-ECs were measured during 24 hours of IFN-gamma treatment. OCR values were normalized for cell density. Data presented as the mean +/- SEM. Multiple comparisons were performed by two-way ANOVA using GraphPad Prism 9. [Time x dose for FCCP interval is ***p*= 0.0018]

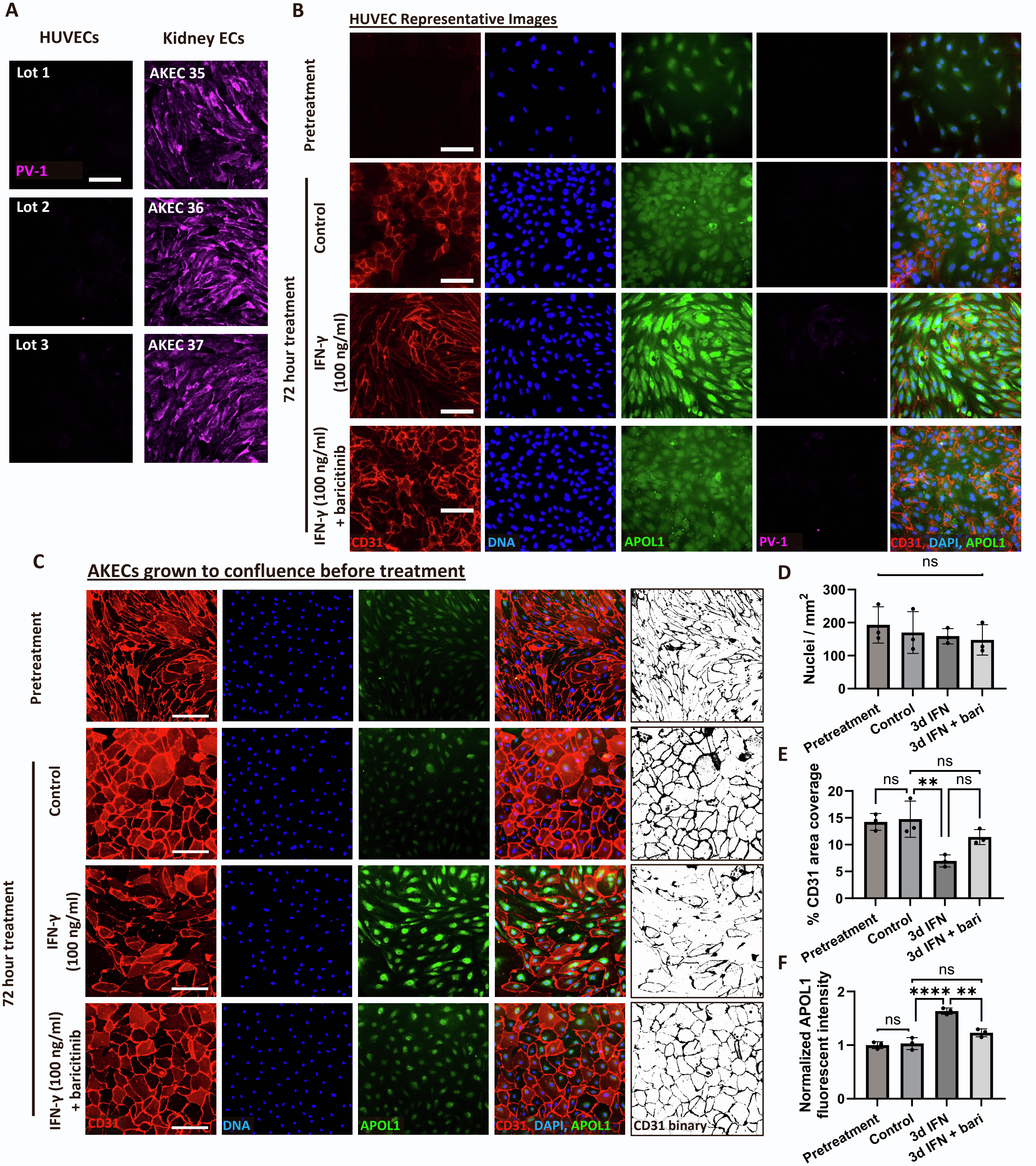


**Figure S5. AKECS grown to confluence demonstrate pronounced decrease in CD31 expression with IFN treatment, Related to Figure 6.** (A) Selective expression of plasmalemmal vesicle–associated protein-1 (PV-1) in primary kidney ECs but not in HUVECs. Representative wide field immunofluorescent images of each HUVEC lot and each kidney donor in control conditions after 72 hour treatments. Scale bar 100 µm. (B) Representative 20x wide field immunofluorescent images of HUVECs 24 hours after plating (pretreatment) and after 72 hour (3d) treatments. Scale bars 100 µm. (C) Representative 10x wide field immunofluorescent images of AKECs immediately after being grown to confluence (pretreatment), and after 72 hour treatments, including CD31 binaries used for image quantification. Scale bars 200 µm. (D, E, F) Automated quantification of (D) average nuclear density, (E) percent CD31+ area coverage and (F) Normalized APOL1 fluorescent intensity in endothelial cell bodies during monolayer culture of primary adult kidney endothelial cells (AKECs, n = 3 independent donors). Mean ± S.D. Significance was calculated using one-way ANOVA with Tukey's multiple comparisons test. $^{**}p<0.01$, $^{****}p<0.0001$ CD31, Cluster of Differentiation 31; PV-1, plasmalemmal vesicle associated protein-1.

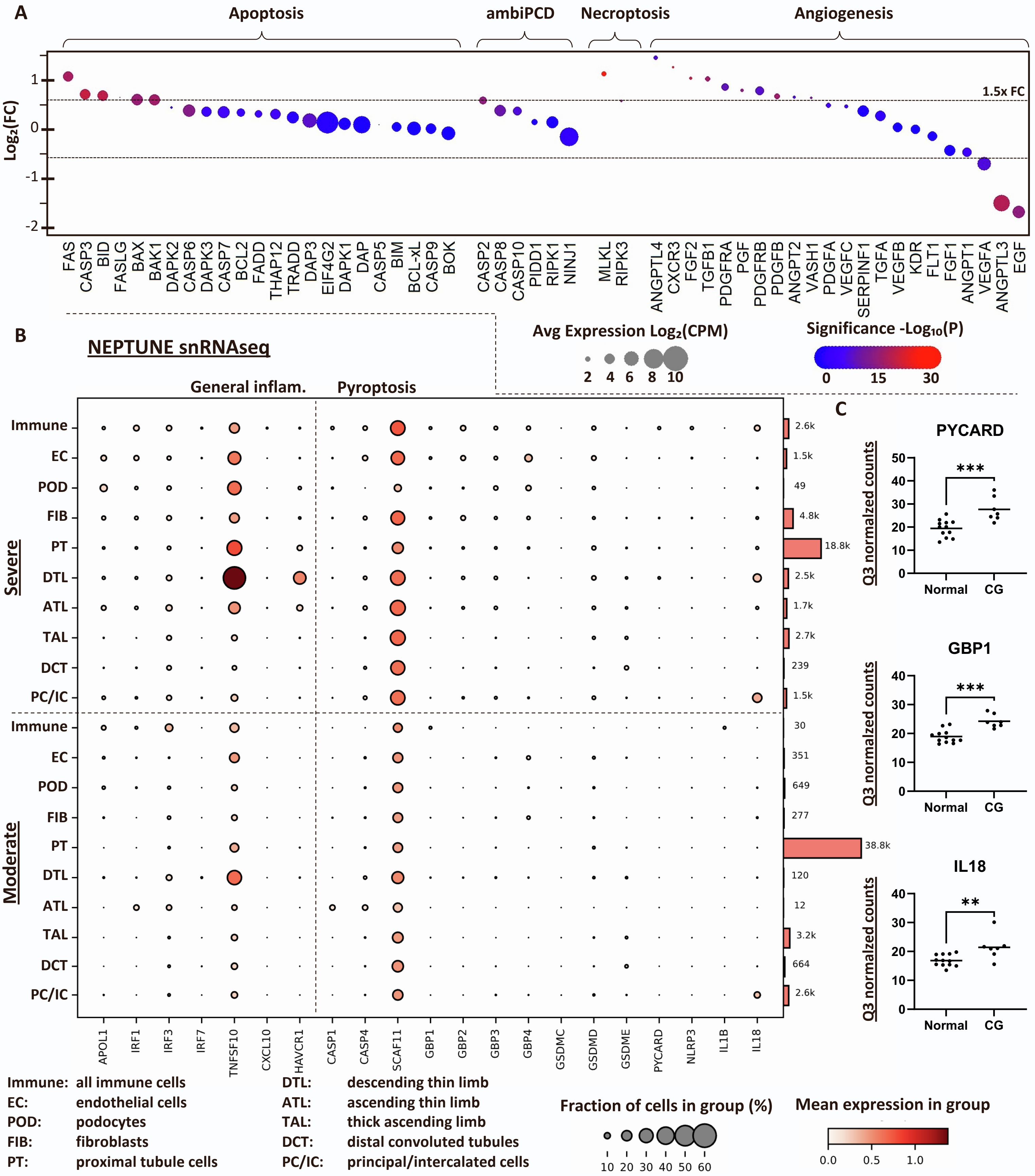


**Figure S6. Individuals with accelerated kidney disease progression and collapsed glomeruli in individuals with FSGS demonstrate upregulation of pyroptosis-associated genes, Related to Figure 7.** (A) Differential gene expression analysis for apoptosis-, necroptosis-, ambiPCD- (ambiguous programmed cell death association), and angiogenesis-associated genes on voom transformed normalized read counts from bulk RNA-seq on biopsies from individuals in the severe compared to moderate cluster, showing fold change (FC), significance (adjusted p-value), and counts per million reads (CPM). Dotted line indicates absolute fold change of 1.5x. (B) snRNA-seq showing upregulation of pyroptosis associated genes in all kidney cell fractions from severe compared to moderate cluster biopsies. (C) Third quartile (Q3) normalized counts for *PYCARD, GBP1*, and *IL18* from collapsing (n=7, 3 patients) and normal (n=12, 3 patients) glomeruli are shown. Each point represents the expression of that gene from GeoMx digital spatial profiling for an individual glomerulus. Mean shown. Significance was calculated using a two-tailed t-test. $^{**}p<0.01$, $^{***}p<0.001$.

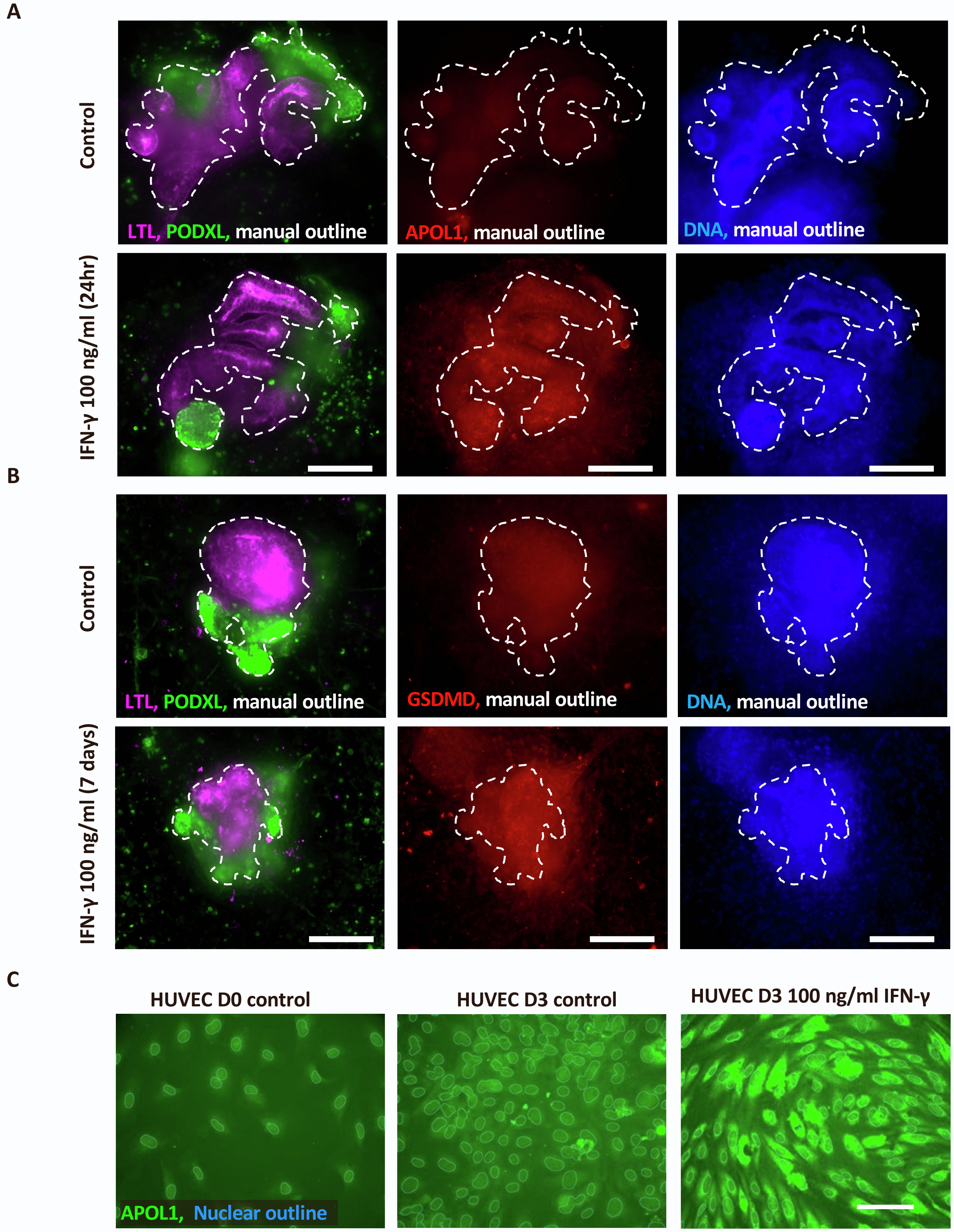


**Figure S7. Representative examples for quantitative fluorescence microscopy analysis in organoids and endothelial monocultures, Related to STAR Methods.** (A, B) Representative maximum intensity projections used for quantitative immunofluorescent image analysis in organoids. Z-stacks were acquired through the full thickness of organoids with 10 µm slice spacing and collapsed into maximum intensity projections to quantify average fluorescent signal of (A) ApolipoproteinL-1 (APOL1) and (B) Gasdermin-D (GSDMD) within organoids. Organoid bodies were manually traced (white) using podocalyxin (PODXL) and Lotus Tetragonolobus Lectin (LTL) to mark podocytes and tubules (respectively). The average fluorescent intensity of the signal of interest within the outline was then measured and normalized to the control condition. Scale bars 100 µm. (C) Automated quantification of APOL1 signal intensity in monolayer endothelial cultures. Representative wide field immunofluorescent images of HUVECs with automatically generated nuclear outlines used for quantification of APOL1 signal intensity in endothelial cells. Scale bar 100 µm.
